# Age-period-cohort analysis of global, regional, and national trends in laryngeal Cancer among older adults, 1990–2021: insights from the 2021 global burden of disease study

**DOI:** 10.3389/fpubh.2025.1547801

**Published:** 2025-03-27

**Authors:** Zhe Sun, Shencheng Liu, Liuqing Zhang, Xiaoyu Li, Xu Chen, Deshang Chen, Shaochun Liu, Jun Wu

**Affiliations:** ^1^Department of Otolaryngology Head and Neck Surgery, The First Affiliated Hospital of Bengbu Medical University, Bengbu, Anhui, China; ^2^Department of Medical Oncology, Harbin Medical University Cancer Hospital, Harbin Medical University, Harbin, Heilongjiang, China

**Keywords:** laryngeal cancer, elderly, global trends, healthcare access, incidence, mortality

## Abstract

**Background:**

Laryngeal cancer poses a significant health burden among older adults, with varying incidence, mortality, and disability-adjusted life years (DALYs) across regions. These disparities highlight the need for targeted interventions and tailored healthcare policies.

**Objectives:**

To analyze global, regional, and national trends in laryngeal cancer incidence, mortality, and DALYs among older adults from 1990 to 2021 using age-period-cohort modeling and to assess the impact of demographic factors on disease burden across socio-developmental index (SDI) regions.

**Materials and methods:**

Using the 2021 Global Burden of Diseases (GBD) database, trends in age-standardized incidence rates (ASIR), age-standardized mortality rates (ASMR), and age-standardized DALYs rates (ASDR) were analyzed, with a focus on the impact of population growth and aging across SDI regions. Age-period-cohort and Joinpoint regression analyses were conducted to identify temporal trends and critical inflection points across SDI quintiles.

**Results:**

From 1990 to 2021, the global ASIR of laryngeal cancer among older adults decreased from 15.16 to 12.25 per 100,000, with significant shifts in trends observed in 1995, 2002, and 2007. The disease burden in lowand middle SDI regions has declined relatively slower compared to high SDI regions, primarily driven by population growth.

**Conclusions and significance:**

Laryngeal cancer trends highlight the need for targeted healthcare interventions. Low and middle SDI areas require improved access to care and prevention strategies, while high SDI regions benefit from personalized, geriatric-focused care.

## Introduction

Laryngeal cancer is a malignant tumor originating in the epithelial cells of the larynx, commonly associated with prolonged smoking, alcohol consumption, and other environmental and genetic factors (1). In 2019, there were 209,000 new cases of laryngeal cancer globally, including 181,000 in men and 28,500 in women. Between 2010 and 2019, new cases increased by 24.7%, while the age-standardized incidence rate declined by 2.5% (2). This paradox—rising case numbers amid falling age-adjusted rates—reflects two competing forces: improved early detection and smoking cessation programs in high-income countries versus population growth and aging in transitioning economies (3). Data from the Surveillance, Epidemiology, and End Results (SEER) program reported 104,991 cases of laryngeal cancer in the United States from 2000 to 2019, with the highest incidence observed in individuals aged 55–69 years (46.71%) (4). Another global epidemiological study indicates that the burden of laryngeal cancer increases with age, peaking after age 65 (5).

Although the global incidence of laryngeal cancer shows a declining trend, the increasing trend of global aging underscores the need to address the burden of laryngeal cancer in older adults. Over the past 70 years, the fertility rate worldwide has fallen by more than half, with most healthy newborns expected to be concentrated in low-income countries by 2,100 (6). This demographic shift implies that middle- and high-income countries will face increasing labor shortages and aging populations, increasing pressure on healthcare and social security systems. Meanwhile, older adults account for 60% of global cancer cases and 70% of cancer-related deaths (7), making the high cancer burden among elderly patients a significant global public health challenge. Despite a high incidence of cancer among the elderly, they have been grossly underrepresented in clinical trials, which are predominantly designed for young, healthy populations. Consequently, evidence-based guidelines tailored to older patients have been notably lacking, potentially resulting in both undertreatment and overtreatment (8). This burden directly impacts the achievement of the United Nations Sustainable Development Goal 3.4 (SDG 3.4), which aims to reduce premature mortality through prevention and treatment of non-communicable diseases (9).

However, research on elderly laryngeal cancer patients—a small group but one with a substantial disease burden—remains limited. An in-depth analysis of temporal trends in laryngeal cancer prevalence among older adults across countries is essential to provide further epidemiological evidence, track advances in disease management, and inform targeted interventions. The 2021 Global Burden of Disease (GBD) study offers a global perspective for analyzing temporal trends, using the latest epidemiological data and methods to generate population-level health metrics (10). The age-period-cohort (APC) model is a commonly used method for analyzing epidemiological data; it separates age effects (risks associated with individual aging), period effects (influences of social environment or medical advancements over time), and cohort effects (unique risks faced by particular birth cohorts), revealing long-term trends in disease prevalence and mortality. The APC model’s strength lies in integrating multidimensional influences, allowing for a deeper understanding of disease changes across periods and age groups. It is particularly well-suited to studies with extensive periods (11).

In this study, we used GBD 2021 data and applied an APC model to systematically explore temporal trends in laryngeal cancer prevalence among older adults at global, regional, and national levels from 1990 to 2021.

## Methods

### Data source

A comprehensive analysis of global health status was provided by the Global Burden of Disease (GBD) 2021 study, with an emphasis on the burden of diseases and injuries as evaluated by incidence, mortality, and disability-adjusted life years (DALYs) (12). An extensive dataset comprising 100,983 data sources was utilized, reflecting a marked enhancement in both breadth and depth relative to previous iterations. DALYs, which combine the years of life lost due to premature mortality with the years lived with disability, were employed to generate a singular measure of the total health loss attributable to diseases and injuries (13). Data were sourced from scientific literature, household surveys, disease registries, and vital registration. For cause-of-death estimates, data from previous rounds were supplemented by 9,248 new sources, and diverse information types, including cancer registries and police records, were incorporated. Corrections were applied to standardize comparisons across age, sex, location, and time by redistributing non-specific codes and excluding sources with excessive garbage codes. A 5% buffer system was implemented to ensure consistency in data inclusion across cycles (14, 15). In addition, the Socio-Demographic Index (SDI) was employed to classify regions by socioeconomic development, thereby facilitating standardized comparisons of health outcomes (10). The SDI, a composite indicator encompassing per capita income, educational attainment, and fertility rates, was used to reflect the overall developmental status of each region (16).

### Analysis of overall temporal trends in incidence, mortality, and DALYs

Data on laryngeal cancer in elderly patients (60 years and above) were initially extracted from the GBD 2021 database, including incidence rates, mortality rates and DALYs. Temporal trends in laryngeal cancer incidence, mortality, and DALYs over the study period were evaluated using age-standardized incidence rates (ASIR), age-standardized mortality rates (ASMR), and age-standardized DALY rates (ASDR) as the primary metrics. Age standardization was performed using the direct standardization method by employing the age adjust direct function from the epitools package in R (17). A standard population, defined as the global standard population estimated by GBD for the year 2021, was used for this purpose (14).

Joinpoint regression analysis was employed to identify significant inflection points in larynx cancer burden trends (1990–2021) using a log-linear model (ln y = βx). The model iteratively partitioned time series into segments with distinct linear trends through a grid search algorithm, testing potential joinpoints across all possible configurations. The mean squared error (MSE) was calculated for each candidate model, and the configuration minimizing MSE was selected as optimal. To determine the optimal number of joinpoints, a Monte Carlo permutation test (4,500 replicates) was applied with a Bonferroni-adjusted significance threshold (*p* < 0.05), allowing a maximum of five joinpoints (kmax = 5) and a minimum of zero (kmin = 0). Sensitivity analyses comparing models with varying joinpoint limits and residual distribution checks ensured robustness. The annual percent change (APC) for each segment was calculated as follows:


$$APC=\left(e^{\beta }-1\right)\times 100$$

Where *β* represents the segment-specific slope from the log-linear regression (18). The average annual percent change (AAPC), summarizing the overall trend from 1990 to 2021, was derived by weighting segment-specific APCs by their interval lengths. Statistical significance was inferred if the 95% confidence interval (CI) excluded zero (19). A positive AAPC indicates an increasing trend, while a negative AAPC indicates a decreasing trend.

The association between the burden of laryngeal cancer and the effects of age, period, and cohort was further examined using an age-period cohort (APC) model. The model accounted for complex interactions among age, period, and cohort, thereby facilitating improved estimation of their contributions to incidence and mortality changes. These effects were decomposed such that age effects were understood to represent changes occurring across the lifespan, period effects were taken to capture external influences affecting the population, and cohort effects were interpreted as reflecting generational differences (20). Multicollinearity among age, period, and cohort was addressed by applying the intrinsic estimator (IE) method based on principal component regression, thereby yielding more robust and interpretable estimates of temporal trends.

### Decomposition analysis

Decomposition analysis was conducted using the Das Gupta method to systematically evaluate temporal shifts in the burden of laryngeal cancer among elderly patients (aged ≥60 years) between 1990 and 2021 (21). This methodology was selected for its capacity to disentangle complex contributions from three interdependent drivers: (1) epidemiological transitions (e.g., changes in incidence, survival, or risk factor prevalence), (2) population growth dynamics, and (3) structural aging of the population (shifts in age distribution). The overall change in disease burden—quantified through age-standardized incidence, mortality, and DALYs rates—was decomposed into additive components attributable to each driver. Epidemiological effects were isolated by holding age-specific rates constant while allowing demographic variables to vary, whereas population growth contributions were calculated by fixing rates and age structures at baseline levels. Age distribution effects were derived by comparing observed demographic shifts against counterfactual scenarios with static population pyramids. Sensitivity analyses were performed to assess robustness to extreme value outliers and model assumptions (22).

## Results

### Global trends

**Figure 1:**
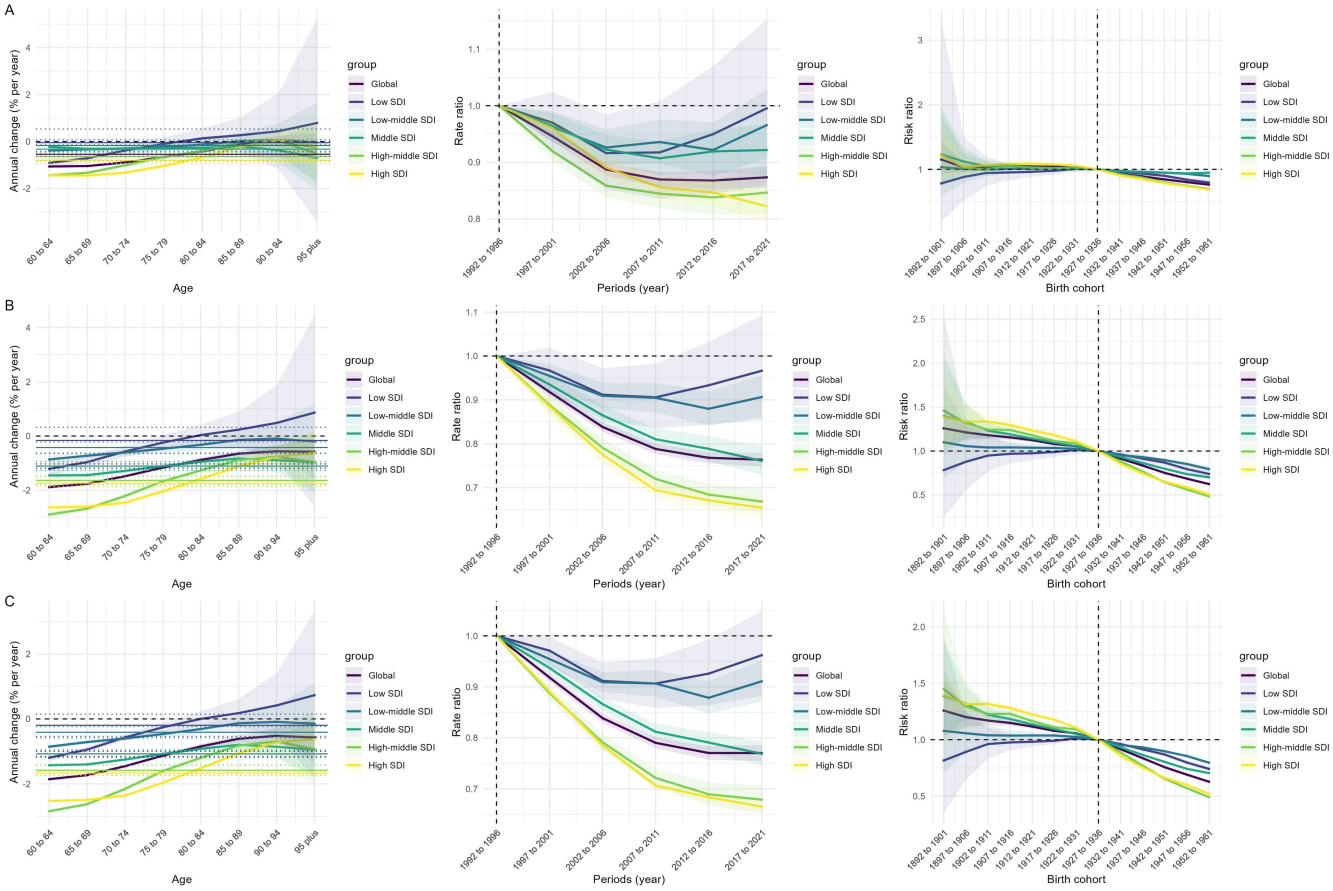
Effects of age, period, and birth cohort on the relative risk of laryngeal cancer ASIR **(A)**, ASMR **(B)**, and ASDR **(C)**. ASIR, age-standardized incidence rate; ASMR, age-standardized mortality rate; ASDR, age-standardized disability-adjusted life years.

As shown in Figure 1, global ASIR, ASMR, and ASDR for laryngeal cancer in older adults increase with age. Specifically, ASIR shows a marked upward trend in those over 70, indicating a substantial disease burden in this population. However, over time, incidence rates show a declining trend. Table 1 indicates that the incidence of laryngeal cancer among older adults worldwide decreased significantly from 1990 to 2021, with an AAPC of −0.68 (95% CI −0.74 to −0.61). In the birth cohort analysis, risk ratios for more recent cohorts approach 1, suggesting that incidence risk for laryngeal cancer has stabilized in recent generations. ASMR similarly increases significantly with age but shows a steady downward trend across periods, especially in recent cohorts. ASDR correlates positively with age, reflecting a high disease burden among older adults, but both time and cohort analyses show a recent decrease in burden rates.

**Table 1:** Incident cases and age-standardized incidence of larynx cancer in adults in 1990 and 2021, and its temporal trends from 1990 to 2021.

Characteristics	1990		2021		1990–2021
	Incident cases (95% UI)	ASIR (95% UI)	Incident cases (95% UI)	ASIR (95% UI)	AAPC (95% CI)
Global	74342.31 (69999.04–78635.75)	15.16 (14.24–16.05)	133795.81 (123281.15–144786.97)	12.25 (11.27–13.25)	−0.68 (−0.74 to −0.61)
High SDI	25495.26 (24142.98–26773.48)	17.7 (16.76–18.6)	35143.22 (32218.39–37398.68)	12.93 (11.91–13.74)	−1.05 (−1.16 to −0.93)
High-middle SDI	22401.35 (20998.6–23888.7)	17.48 (16.34–18.67)	34536.61 (30552.5–38730.86)	13.4 (11.85–15.02)	−0.82 (−1.02 to −0.63)
Low SDI	3077.35 (2420.01–3837.86)	11.92 (9.36–14.88)	5964.86 (5019.41–7025.12)	10.63 (8.94–12.51)	−0.29 (−0.41 to −0.17)
Low-middle SDI	9610.78 (8074.64–11401.54)	13.77 (11.54–16.36)	21870.42 (19334.78–25031.67)	12.78 (11.28–14.64)	−0.18 (−0.46–0.09)
Middle SDI	13653.01 (12263.02–14887.47)	11.6 (10.39–12.64)	36106.74 (31845.66–40953.5)	10.95 (9.64–12.41)	−0.18 (−0.32 to −0.04)
Region					
Andean Latin America	175.73 (142.34–214.3)	7.58 (6.13–9.26)	348.11 (257.5–458.23)	4.88 (3.61–6.42)	−1.38 (−1.94 to −0.83)
Australasia	336.58 (291.95–390.67)	10.82 (9.38–12.56)	425.05 (349.56–506.61)	6.02 (4.96–7.18)	−1.94 (−2.53 to −1.34)
Caribbean	658.85 (578.36–752.18)	20.61 (18.08–23.55)	1475.29 (1203.87–1796.96)	22 (17.95–26.8)	0.24 (−0.47–0.95)
Central Asia	855.47 (800.71–911.12)	14.56 (13.61–15.52)	783.46 (695.75–874.59)	7.75 (6.88–8.65)	−2.17 (−2.34 to −1.99)
Central Europe	3863.9 (3612.03–4133.75)	19.22 (17.94–20.57)	6119.45 (5503.79–6775.84)	20.67 (18.59–22.89)	0.25 (0.07–0.43)
Central Latin America	1287.83 (1211.88–1361.24)	13.88 (13.02–14.68)	2239.34 (1937.87–2570.24)	7.35 (6.36–8.43)	−2.11 (−2.57 to −1.65)
Central Sub-Saharan Africa	223.56 (150.03–312.58)	9.1 (6.17–12.74)	452.29 (319.98–618.71)	7.82 (5.52–10.77)	−0.49 (−0.65 to −0.33)
East Asia	9878.12 (8041.87–11662.44)	9.81 (8–11.55)	27966.44 (21854.92–35423.11)	10.05 (7.87–12.7)	0.04 (−0.11–0.2)
Eastern Europe	6402.44 (6053.45–6780.55)	16.62 (15.7–17.62)	6496.25 (5753.57–7263.59)	13.22 (11.71–14.77)	−0.68 (−1.21 to −0.14)
Eastern Sub-Saharan Africa	765.79 (598.3–942.67)	8.98 (7.02–11.04)	1281.18 (1021.66–1611.65)	6.94 (5.53–8.69)	−0.82 (−0.88 to −0.76)
High-income Asia Pacific	3165.33 (2779.26–3547.45)	12.48 (10.95–13.98)	5149.95 (4289.57–5961.85)	8.36 (6.99–9.69)	−1.32 (−1.99 to −0.64)
High-income North America	9526.48 (9006.8–9951.08)	20.69 (19.58–21.61)	13424.06 (12417.63–14186.83)	15.22 (14.09–16.07)	−1.05 (−1.21 to −0.88)
North Africa and Middle East	2864.81 (2333.08–3540.29)	15.16 (12.29–18.85)	7482.8 (6301.15–8794.09)	14.76 (12.4–17.36)	−0.06 (−0.13–0)
Oceania	10.9 (7.88–15.13)	3.74 (2.72–5.2)	24.64 (17.73–34.47)	3.39 (2.43–4.78)	−0.29 (−0.38 to −0.21)
South Asia	11181.56 (9202.64–13400.99)	17.2 (14.08–20.66)	27050.63 (23366.42–31269.84)	15.13 (13.06–17.5)	−0.36 (−0.58 to −0.14)
Southeast Asia	2334.42 (1957.44–2748.77)	8.17 (6.83–9.64)	6692.83 (5580.91–8101.96)	8.57 (7.13–10.4)	0.15 (0.06–0.24)
Southern Latin America	1155.24 (986.34–1337.49)	19.36 (16.52–22.42)	1358.03 (1137.95–1606.42)	12.09 (10.14–14.31)	−1.46 (−1.71 to −1.2)
Southern Sub-Saharan Africa	312.96 (250.79–419.14)	9.82 (7.87–13.11)	665.81 (581.01–754.53)	9.53 (8.3–10.81)	−0.12 (−0.37–0.14)
Tropical Latin America	1673.25 (1545.17–1802.82)	15.48 (14.24–16.7)	4656.89 (4177.16–5120.31)	14.38 (12.88–15.81)	−0.18 (−0.43–0.08)
Western Europe	17016.85 (15785.99–18265.11)	22.57 (20.94–24.23)	18300.15 (16277.75–20176.95)	15.77 (14.1–17.38)	−1.13 (−1.34 to −0.93)
Western Sub-Saharan Africa	652.22 (515.33–812.47)	6.39 (5.08–7.92)	1403.15 (1116.27–1693.33)	6.67 (5.33–8.01)	0.14 (0.1–0.18)

ASIR, age-standardized incidence rate; AAPC, average annual percentage change; UI, uncertainty interval; CI, confidence interval.

**Figure 2:**
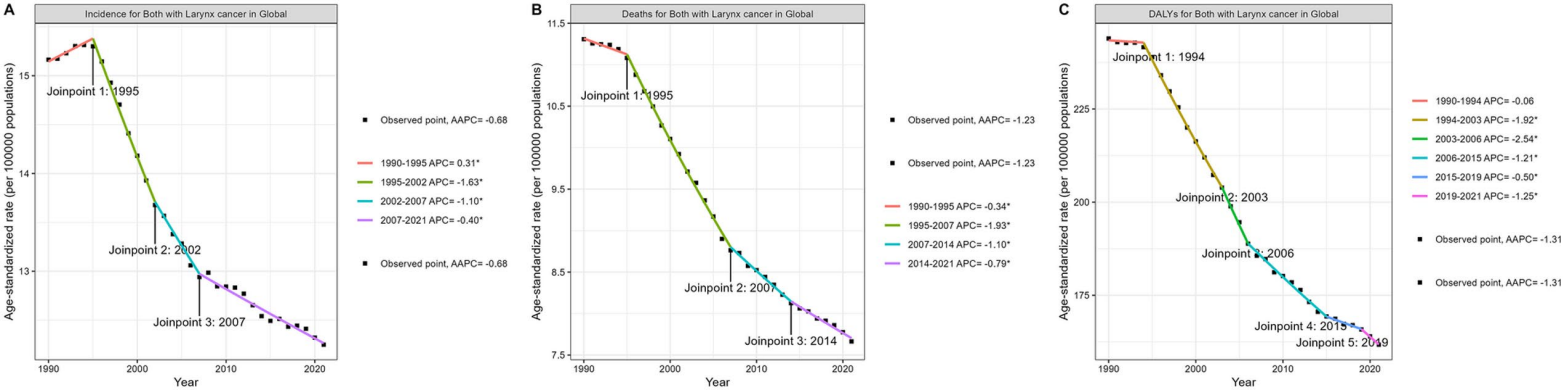
Joinpoint regression analysis of ASIR **(A)**, ASMR **(B)**, and ASDR **(C)**. ASIR, age-standardized incidence rate; ASMR, age-standardized mortality rate; ASDR, age-standardized disability-adjusted life years.

According to the global Joinpoint analysis results for laryngeal cancer in Figure 2, ASIR, ASMR, and ASDR have all shown significant declines since 1990, with varying APCs over different periods. Figure 2A shows that ASIR for laryngeal cancer underwent three fundamental shifts between 1990 and 2021: from 1990 to 1995, the APC was 0.31% (a slight increase); from 1995 to 2002, the APC dropped sharply to −1.63%; from 2002 to 2007, it declined further to −1.10%; and from 2007 to 2021, the decline slowed to −0.40%, with an overall AAPC of −0.68%. Figure 2B shows that ASMR also declined during the same period, with an APC of −0.34% from 1990 to 1995, accelerating to −1.93% from 1995 to 2007, then decreasing slightly to −1.10% from 2007 to 2014, and further slowing to −0.79% from 2014 to 2021, with an overall AAPC of −1.23%. Figure 2C shows that ASDR also declined significantly, with an APC of −0.06% from 1990 to 1994, accelerating to −1.92% from 1994 to 2003, dropping further to −2.54% from 2003 to 2006, and slowing to −0.50% and − 1.25% from 2015 to 2019 and 2019 to 2021, respectively, with an overall AAPC of −1.31%.

### Trends by SDI quintile

As depicted in Figure 1, notable differences in laryngeal cancer burden trends have been observed across various SDI regions. Although a relatively high ASIR is noted among older adults in low SDI regions, incidence rates have declined in recent years. In parallel, ASMR and ASDR have gradually decreased, thereby indicating an increasing effectiveness of health interventions. In low-middle SDI regions, ASIR has been found to increase with age, albeit with a recent decline, while ASMR and ASDR similarly increase with age but have significantly decreased in recent years, reflecting improvements in health resources. In middle SDI regions, ASIR is noted to rise with age but has declined over time, with a concomitant decrease in cohort risk. In high-middle SDI regions, moderate age-related increases in ASIR have been recorded; overall incidence rates have been markedly reduced over time, with cohort risk substantially diminished in recent birth cohorts. In high SDI regions, slight variations in ASIR with age have been detected, alongside steadily decreasing incidence rates over time and lower cohort risks among recent generations.

Table 1 indicates that the most significant decline in laryngeal cancer incidence among older adults from 1990 to 2021 was observed in high SDI regions (AAPC −1.05 [95% CI −1.16 to −0.93]). Notably, in 1990, the lowest ASIR was documented in middle SDI regions at 11.6 per 100,000 population (95% UI 10.39–12.64), whereas in 2021, the lowest ASIR was recorded in low SDI regions at 10.63 per 100,000 population (95% UI 8.94–12.51). Conversely, in 1990, the highest ASIR was noted in high SDI regions at 17.7 per 100,000 population (95% UI 16.76–18.6), while in 2021, the highest ASIR was observed in high-middle SDI regions at 13.4 per 100,000 population (95% UI 11.85–15.02). Supplementary Figure S1 illustrates the relationship between SDI and all-cause mortality across 204 countries or territories, while Supplementary Figure S3 presents the Joinpoint regression analysis across the five SDI regions.

### Trends by 21 regions and 204 countries and territories

**Figure 3:**
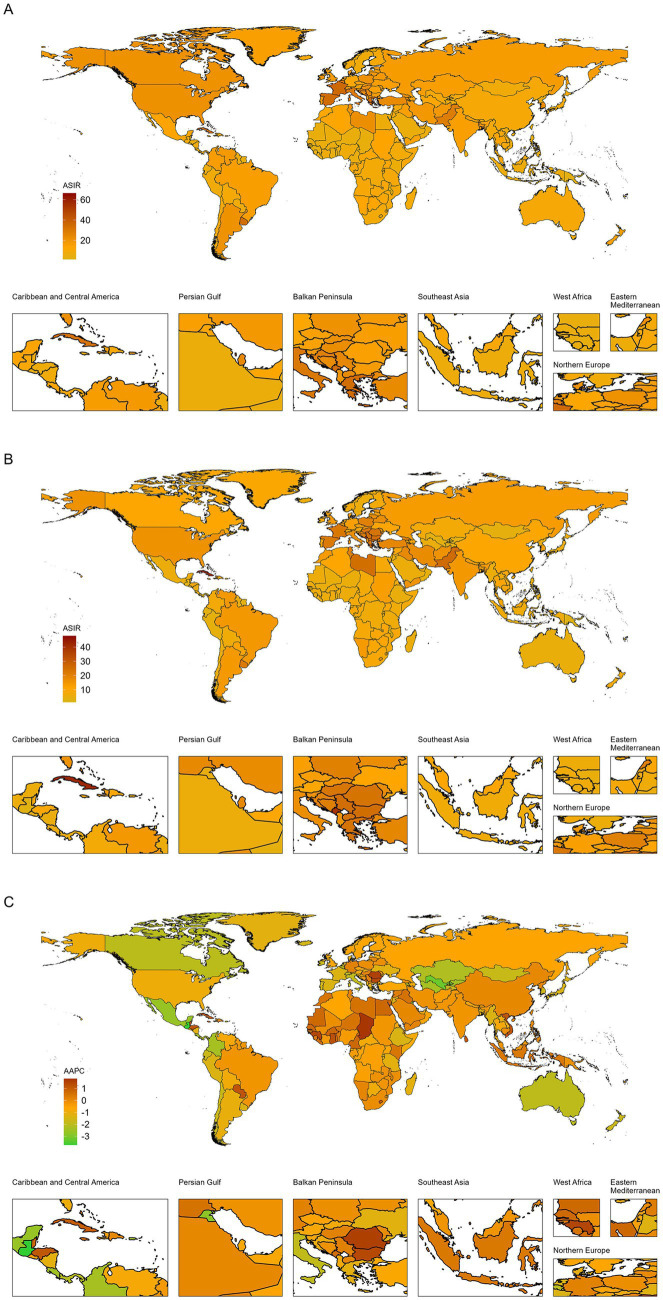
Global map of ASIR of laryngeal cancer in 1990 **(A)** and 2021 **(B)** and its AAPC **(C)**. ASIR, age-standardized incidence rate; AAPC, average annual percentage change.

As shown in Tables 1 and Supplementary Tables S1–S3, the Caribbean had the highest ASIR, ASMR, and ASDR among regions in 2021. From 1990 to 2021, Central Europe was the only region with a slight increase in ASIR, rising from 19.2 per 100,000 population (95% uncertainty interval [UI] 17.94–20.57) in 1990 to 20.67 per 100,000 population (95% UI 18.59–22.89) in 2021 (AAPC 0.25 [95% CI 0.07 to 0.43]). At the country and territory level, Montenegro had the highest ASIR in 2021. Guatemala showed the most significant ASIR decrease from 1990 to 2021, dropping from 10.14 per 100,000 population (95% UI 9.02–11.32) in 1990 to 3.22 per 100,000 population (95% UI 2.63–3.89) in 2021 (AAPC −3.73 [95% CI −4.68 to −2.77]). Figure 3 illustrates the differences across countries and territories in 1990 and 2021, along with corresponding AAPC values.

### Decomposition analysis

**Figure 4:**
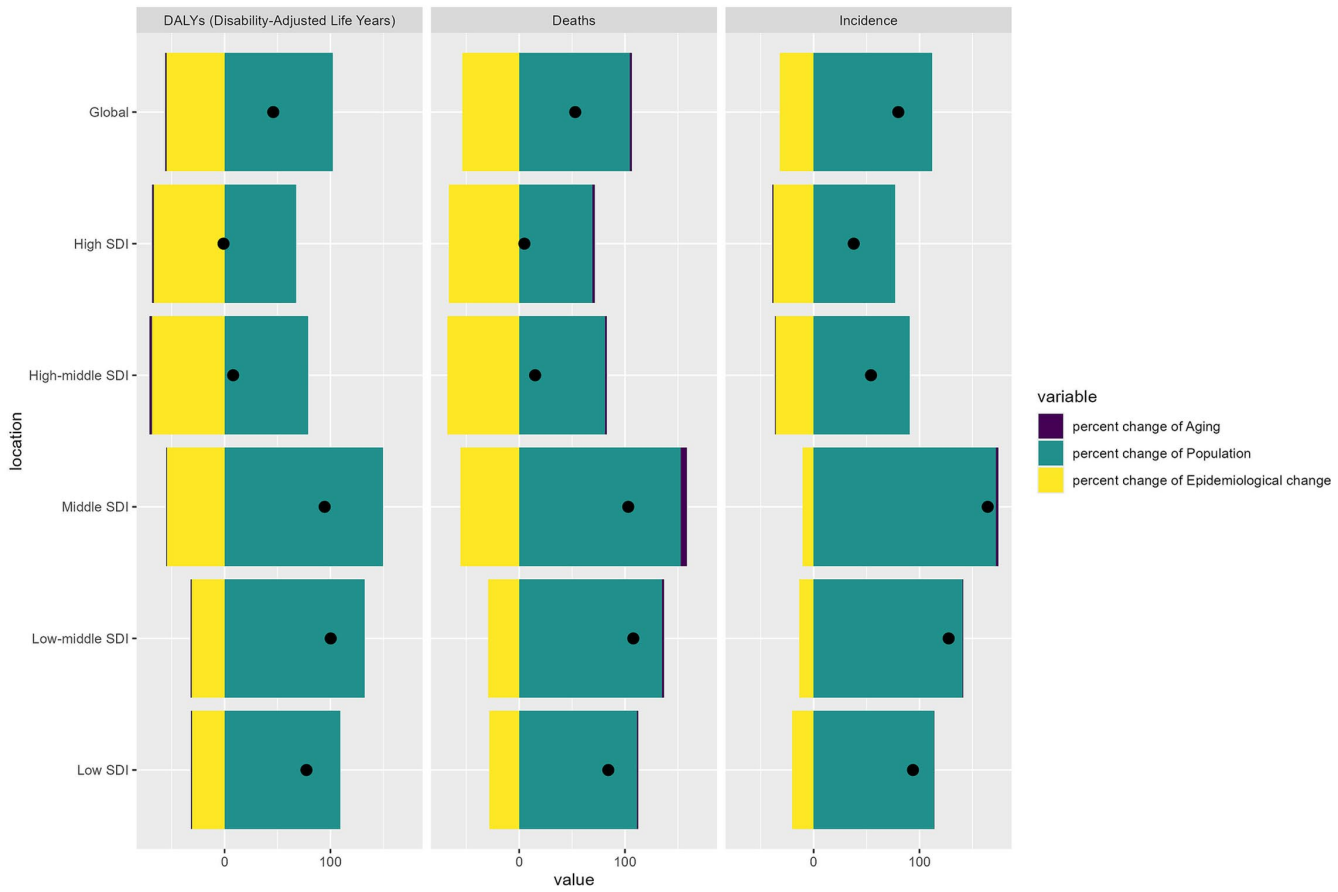
Decomposition analysis of incidence, mortality, and DALYs. DALYs, disability-adjusted life years.

Figure 4 illustrates the decomposition analysis of the burden of laryngeal cancer among elderly patients worldwide, segmented by SDI levels. Population growth emerges as the dominant contributor to the increase in incidence, mortality, and DALYs across all SDI regions, particularly in low and middle SDI regions, where it accounts for the largest share of the burden. However, the effects of aging and epidemiological changes are more pronounced in high SDI areas. Aging significantly contributes to the burden, reflecting the increasing proportion of elderly individuals within these populations. Additionally, epidemiological changes—encompassing shifts in risk factors, healthcare access, and disease management—are particularly significant in high SDI and high-middle SDI regions. This suggests that changes in disease patterns and healthcare interventions play an increasingly vital role. Consequently, there is a pressing need for targeted strategies in low SDI and middle SDI regions to manage the impact of population growth. In contrast, in higher SDI regions, interventions addressing aging and evolving epidemiological trends are crucial for mitigating the burden of laryngeal cancer among the elderly.

## Discussion

This study provides a comprehensive analysis of the temporal trends in laryngeal cancer incidence, mortality, and DALYs among elderly patients from 1990 to 2021, utilizing data from the GBD study. Our findings highlight the complex interplay between demographic factors, health interventions, and epidemiological trends that shape the burden of laryngeal cancer, particularly among older adults.

Although the absolute numbers of incidence, mortality, and DALYs for laryngeal cancer have risen from 1990 to 2021—primarily due to the marked growth in the elderly population—ASIR, ASMR, and ASDR have shown a decline over this period. This observation is consistent with previous findings from the GBD 2019 study encompassing all age groups (23). It suggests that improvements in healthcare quality and preventive interventions have effectively mitigated the overall burden of laryngeal cancer. Notably, the joinpoint analysis reveals that while incidence in older adults increased from 1990 to 1995, subsequent years witnessed marked declines in incidence, mortality, and DALYs. Such trends may be attributed, in part, to the introduction and gradual global adoption of electronic laryngoscopy in the early 1990s (24), followed by subsequent advances in surgical techniques, radiotherapy, chemotherapy, and targeted therapies (25, 26).

Moreover, the birth cohort analysis indicates that the risk of laryngeal cancer has stabilized among recent generations, suggesting that the factors driving incidence are modifiable via effective public health strategies. In this context, targeted anti-tobacco initiatives and lifestyle interventions appear particularly promising. Smoking remains a significant risk factor for laryngeal cancer (27), and over the past three decades, a range of anti-smoking campaigns have been implemented worldwide. For example, China amended its Advertising Law in 2015 to prohibit promoting tobacco products (28). Similarly, the implementation of tobacco control policies in the United States has demonstrated significant efficacy; data indicate that the incidence and mortality rates of smoking-related cancers decreased between 2004 and 2013, a trend closely associated with these measures (29). Strengthening these preventive efforts could prove crucial in reducing the future burden of laryngeal cancer.

The findings reveal substantial disparities in laryngeal cancer burden trends across different SDI regions. Recent declines in ASIR, ASMR, and ASDR in low SDI areas indicate progress in health interventions, likely reflecting improvements in healthcare access and public health awareness. These downward trends suggest that even modest advancements in medical resources can significantly improve disease outcomes in low SDI environments. Similarly, in low-middle SDI regions, reductions in incidence, mortality, and disability rates may be attributed to gradual healthcare infrastructure and resource enhancements. However, the ongoing aging burden in these regions underscores the need for continued healthcare interventions and resources targeting the elderly population. In middle and high-middle SDI regions, the declining ASIR, ASMR, and ASDR suggest that public health efforts yield positive results, particularly in tobacco cessation programs and improved healthcare access. High SDI regions, with steadily decreasing incidence rates, demonstrate the impact of well-established healthcare systems and public health policies, including widespread tobacco control measures and early detection strategies, contributing to reduced laryngeal cancer burden. The notable decline in AAPC in high-SDI areas further supports the role of robust health systems in reducing cancer burdens.

Nevertheless, regional disparities persist. Areas such as the Caribbean and Central Europe continue to exhibit elevated ASIR and ASMR values, highlighting the need for tailored strategies that account for local SDI levels and unique regional challenges. A recent study underscored the growing prevalence of youth smoking in certain Central European countries (30), which may portend adverse long-term implications for laryngeal cancer incidence among older populations. At the national level, these disparities underscore the influence of local public health policies and healthcare capacities on cancer outcomes. For instance, the marked decrease in mortality observed in Guatemala attests to the benefits of targeted interventions. In contrast, persistent mortality in Montenegro indicates a pressing need for public health improvements. Moreover, while reductions in smoking prevalence have been documented across Latin America and the Caribbean since 2000, alcohol consumption remains high, further complicating the regional risk landscape.

Consequently, continued efforts in these regions should focus on reducing alcohol-related risks while sustaining anti-tobacco initiatives. While the overall populations of these countries are relatively small, which may introduce variability in data estimates, these national findings nonetheless emphasize the importance of region-specific strategies in optimizing global laryngeal cancer prevention and control. Tailored approaches that address local health behaviors and resource limitations will be essential to effectively reduce the burden of laryngeal cancer.

The decomposition analysis provides further insight into the drivers underlying these trends. In low and middle SDI regions, population growth emerges as the primary factor driving the rising incidence, mortality, and DALYs associated with laryngeal cancer. This finding underscores the urgent need for public health interventions that address the impact of population increases, such as expanding access to healthcare services, developing preventive strategies, and launching public awareness campaigns targeting risk factors like smoking and alcohol consumption. For example, tobacco control measures implemented in countries such as India and Brazil have contributed to reductions in smoking rates (31, 32), which may, in turn, help lower laryngeal cancer incidence among older adults. Nevertheless, these regions face a significant burden mainly due to population growth.

In contrast, high SDI regions are increasingly affected by aging populations and evolving epidemiological factors, including advances in healthcare and early detection technologies. For instance, the widespread adoption of advanced diagnostic tools such as electronic laryngoscopy and imaging methods in countries like the United States and Japan has improved diagnostic accuracy, contributing to lower mortality rates. Additionally, smoking reduction measures, like the US Family Smoking Prevention and Tobacco Control Act of 2009 (33), have helped curb incidence rates. This shift calls for comprehensive health policies addressing population dynamics and changing disease risk factors.

As healthcare systems in high SDI regions continue to adapt to an aging demographic, there is an increasing imperative to implement personalized medicine approaches and comprehensive geriatric assessments to meet the specific needs of older adults at high risk for laryngeal cancer. The underrepresentation of this age group in clinical research, as highlighted in the Institute of Medicine’s 2013 report on delivering high-quality cancer care (34), further underscores the necessity for tailored, evidence-based strategies to optimize clinical outcomes and improve quality of life for elderly patients worldwide.

Despite these valuable insights, several limitations must be acknowledged. The reliance on secondary data sources, such as the GBD study, may introduce variability and inconsistencies stemming from differences in data collection methods and reporting practices across countries. Furthermore, while multiple factors influencing laryngeal cancer trends were examined, other potential confounders—including socioeconomic status, environmental exposures, and genetic predispositions—were not fully explored, potentially limiting the comprehensiveness of our findings. Finally, the broad temporal scope of this analysis may obscure more nuanced changes within specific populations or regions, and regional variations in healthcare access, cultural practices, and risk factor prevalence may limit the generalizability of the results. Future research should address these limitations by incorporating more granular data and evaluating the impact of local interventions on laryngeal cancer trends.

## Conclusion

In summary, this study underscores the continued decline in the burden of laryngeal cancer among elderly populations from 1990 to 2021. Although global ASIR trends have shown a decrease, the aging population highlights the need for sustained public health efforts explicitly tailored for older adults. The marked regional disparities across different SDI levels underline the need for context-specific strategies, especially in low and middle SDI regions, where population growth drives the laryngeal cancer burden. Meanwhile, high SDI regions are increasingly impacted by aging and shifting epidemiological patterns, requiring adaptive healthcare strategies to address these evolving challenges. Our findings highlight the importance of ongoing surveillance and proactive interventions aimed at reducing the burden of laryngeal cancer in older populations, ultimately improving their outcomes and quality of life.

## Data Availability

Publicly available datasets were analyzed in this study. This data can be found at: https://ghdx.healthdata.org/gbd-2021.
