# Supplementary material for: Age-period-cohort analysis of global, regional, and national trends in laryngeal Cancer among older adults, 1990–2021: insights from the 2021 global burden of disease study
Source: Front Public Health. 2025 Mar 27;13:1547801. doi: 10.3389/fpubh.2025.1547801 (PMC11983630; doi:10.3389/fpubh.2025.1547801)
Supplement: Supplementary file 1 [file Data_Sheet_1.pdf]

Supplementary Materials

Theoretical Framework 2

Figure S1. The relationship between the SDI and larynx cancer mortality in older adults across 204 countries and territories in 2021. 4

Figure S2. The joinpoint regression analysis of ASIR, ASMR, and ASDR across the five SDI regions. 5

Figure S3. AAPC in global and different SDI locations. 5

Figure S4. Global map of ASMR of laryngeal cancer in 1990(A) and 2021(B) and its AAPC(C). 7

Figure S5. Global map of ASDR of laryngeal cancer in 1990(A) and 2021(B) and its AAPC(C). 9

Table S1. The incident cases and ASIR of larynx cancer among old adults in 1990 and 2021, along with the AAPC from 1990 to 2021, across 204 countries and territories. 10

Table S2. The death cases and ASMR of older adults with larynx cancer in 1990 and 2021, and its AAPC from 1990 to 2021 16

Table S3. The DALYs and ASDR of older adults with larynx cancer in 1990 and 2021, and its AAPC from 1990 to 2021 24

Table S4. Changes in incident number according to population-level determinants and causes from 1990 to 2021. 31

Table S5. Changes in death number according to population-level determinants and causes from 1990 to 2021. 32

Table S6. Changes in DALYs number according to population-level determinants and causes from 1990 to 2021. 33

Theoretical Framework

This study employs an integrated analytical framework that synthesizes three complementary perspectives to investigate laryngeal cancer epidemiology in aging populations. At its core lies the Global Burden of Disease (GBD) study's macro-epidemiological approach, which quantifies health impacts through standardized metrics like Disability-Adjusted Life Years (DALYs) and Years Lived with Disability (YLDs). By integrating multi-source data—from cancer registries to environmental exposure assessments—the GBD framework enables cross-regional comparisons of disease burden while accounting for sociodemographic disparities. For instance, the GBD 2021 analysis identified particulate matter pollution and metabolic risks as leading contributors to global health deterioration, offering actionable insights for policymakers.[1] This macro-level perspective is uniquely suited to laryngeal cancer research, where regional variations in air quality and aging demographics critically shape disease patterns.

To unravel temporal trends, the Age-Period-Cohort (APC) model dissects disease burden into three dimensions: age effects (cumulative biological risks from lifelong exposures like smoking), period effects (time-bound influences such as advancements in targeted therapies or tobacco control laws), and cohort effects (generational shifts in risk behaviors, such as dietary changes or occupational carcinogen exposures)[2]. For example, rising laryngeal cancer incidence among older adults may reflect age-related immune decline, while declining mortality in high-income countries aligns with period-specific innovations in minimally invasive surgeries. The APC model's strength lies in distinguishing population aging (an age effect) from healthcare advancements (a period effect), thereby clarifying their respective roles in shaping epidemiological transitions.

Complementing these perspectives, the Socio-Ecological Health Model elucidates how multilevel interactions—spanning individual behaviors such as alcohol consumption, community environments including industrial pollution hotspots, and national policies exemplified by PM2.5 emission standards—collectively drive health disparities. In low-resource settings, limited access to cancer screening, a community-level barrier, exacerbates risks posed by individual smoking habits, whereas high-income regions leverage policy interventions such as HPV vaccination programs to mitigate aging-related burdens[3]. This model bridges the GBD's macro-level stratification and the APC's temporal analysis, revealing how structural inequities amplify or attenuate disease risks across generations.

The integration of these frameworks aligns with the WHO's life-course epidemiology paradigm, offering actionable pathways for age-friendly cancer control. For instance, GBD-derived risk mappings can prioritize regions for pollution control (addressing period effects), while APC-driven cohort analyses inform tailored smoking cessation programs for high-risk birth generations. Simultaneously, socio-ecological insights guide community-level screening initiatives in industrial zones with elevated asbestos exposure histories. By unifying population-level metrics, temporal trend decomposition, and contextual risk profiling, this

framework advances precision public health strategies that address both biological aging and systemic inequities—a critical step toward achieving global targets like SDG 3.4[4].

References

[1] Roth GA, Mensah GA, Johnson CO, et al. Global burden of cardiovascular diseases and risk factors, 1990–2019: update from the GBD 2019 study [J]. 2020, 76(25) : 2982-3021.

[2] Kupper LL, Janis JM, Karmous A, et al. Statistical age-period-cohort analysis: a review and critique [J]. 1985, 38(10) : 811-30.

[3] Kilanowski JFJJOA. Breadth of the socio-ecological model [J]. 2017, 22(4) : 295-7.

[4] Bennett JE, Kontis V, Mathers CD, et al. NCD Countdown 2030: pathways to achieving Sustainable Development Goal target 3.4 [J]. 2020, 396(10255) : 918-34.

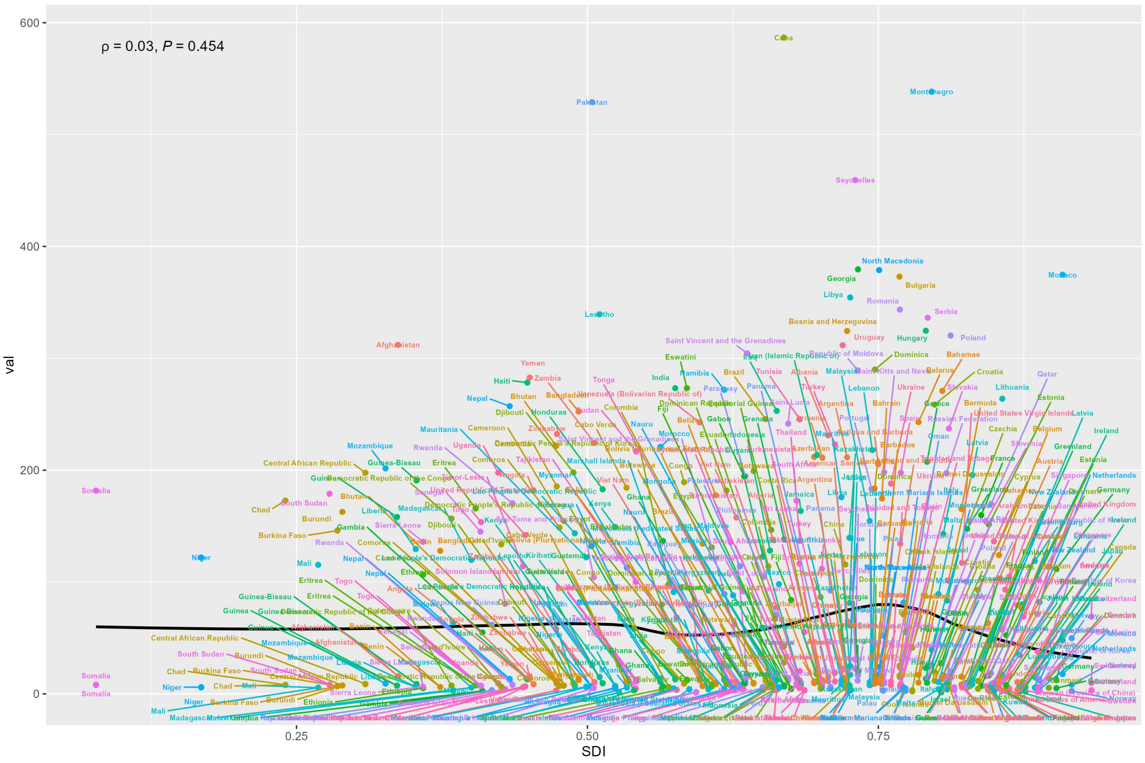

Figure S1. The relationship between the SDI and larynx cancer mortality in older adults across 204 countries and territories in 2021.

Abbreviations: SDI= Socio-demographic Index.

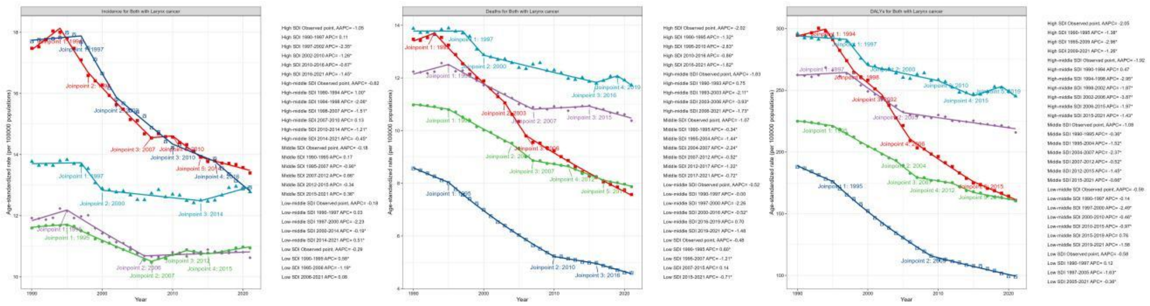

Figure S2. The joinpoint regression analysis of ASIR, ASMR, and ASDR across the five SDI regions.

Abbreviations: SDI: Sociodemographic Index, ASIR: age-standardised incidence rate, ASMR: age-standardised mortality rate, ASDR: age-standardised disability-adjusted life years rate.

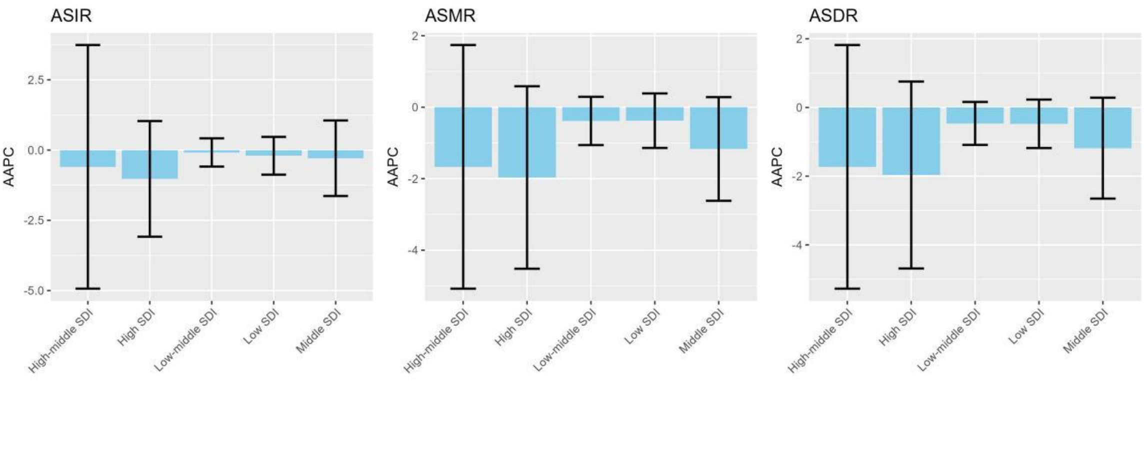

Figure S3. AAPC in global and different SDI locations.

Abbreviations: SDI: Sociodemographic Index, ASIR: age-standardised incidence rate, ASMR: age-standardised mortality rate, ASDR: age-standardised disability-adjusted life years rate.

A

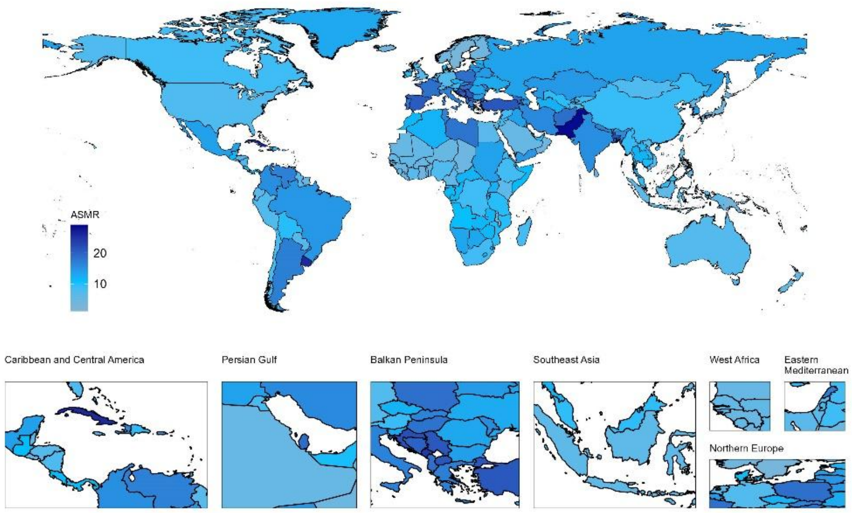
ASMR
20
10
Caribbean and Central America
Persian Gulf
Balkan Peninsula
Southeast Asia
West Africa
Eastern Mediterranean
Northern Europe

B

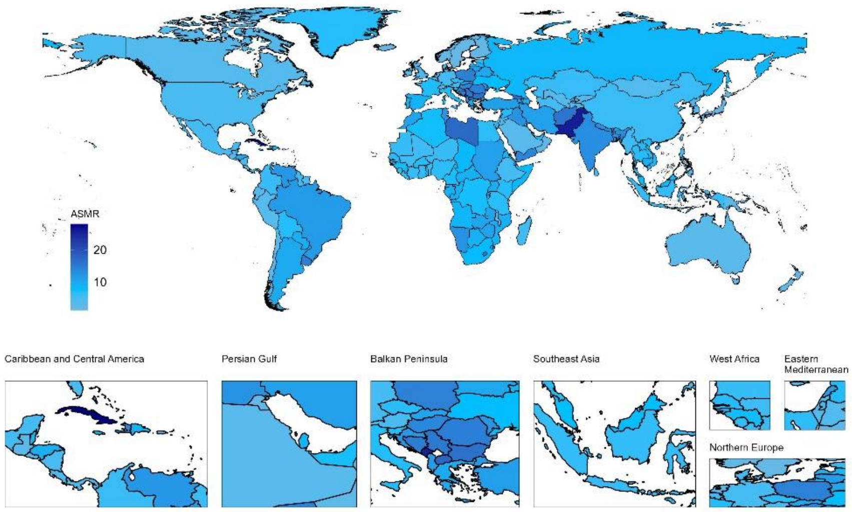
ASMR
20
10
Caribbean and Central America
Persian Gulf
Balkan Peninsula
Southeast Asia
West Africa
Eastern Mediterranean
Northern Europe

C

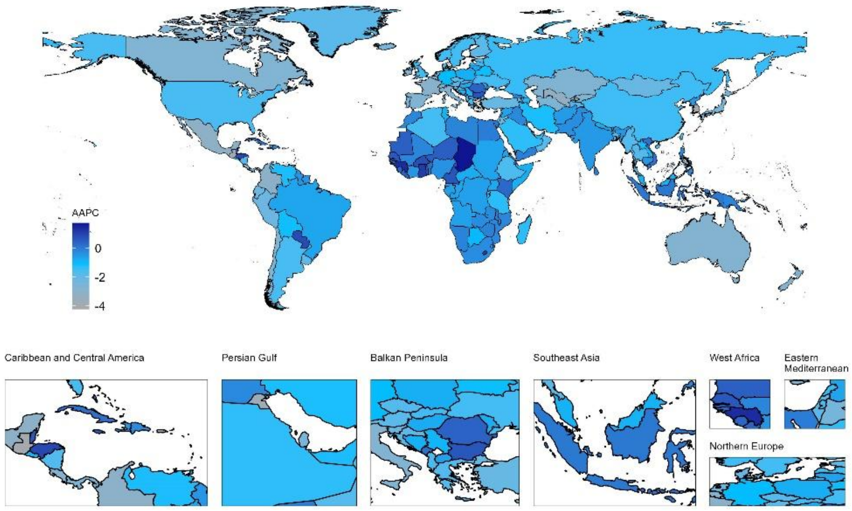
AAPC
0
-2
-4
Caribbean and Central America
Persian Gulf
Balkan Peninsula
Southeast Asia
West Africa
Eastern Mediterranean
Northern Europe

Figure S4. Global map of ASMR of laryngeal cancer in 1990(A) and 2021(B) and its AAPC(C).

Abbreviations: SDI: Sociodemographic Index, ASIR: age-standardised incidence rate, ASMR: age-standardised mortality rate, ASDR: age-standardised disability-adjusted life years rate.

A

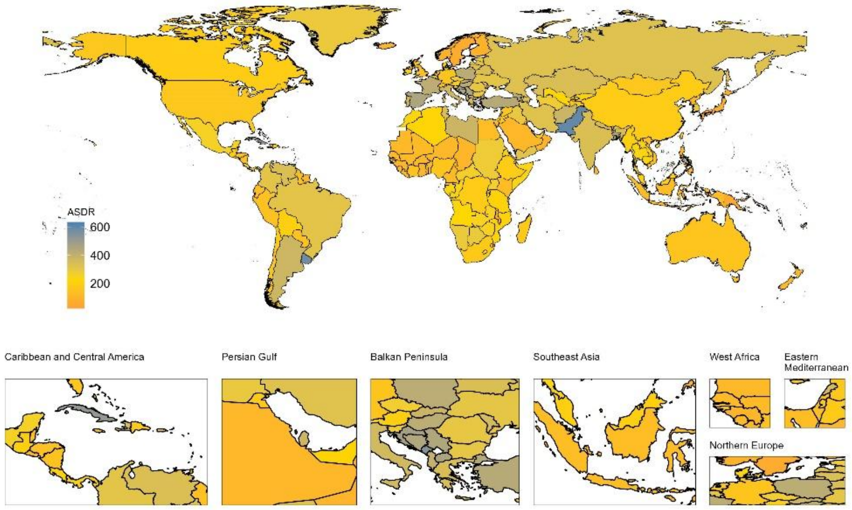
ASDR
600
400
200
Caribbean and Central America
Persian Gulf
Balkan Peninsula
Southeast Asia
West Africa
Eastern Mediterranean
Northern Europe

B

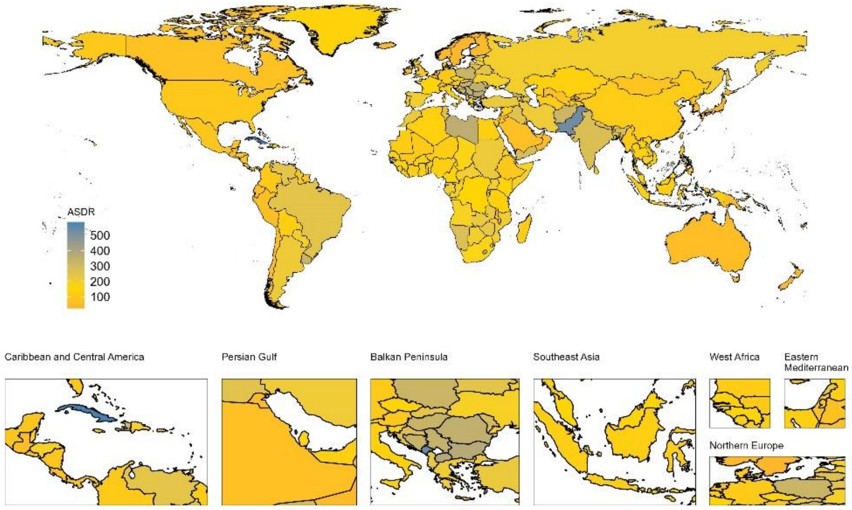
ASDR
500
400
300
200
100
Caribbean and Central America
Persian Gulf
Balkan Peninsula
Southeast Asia
West Africa
Eastern Mediterranean
Northern Europe

C

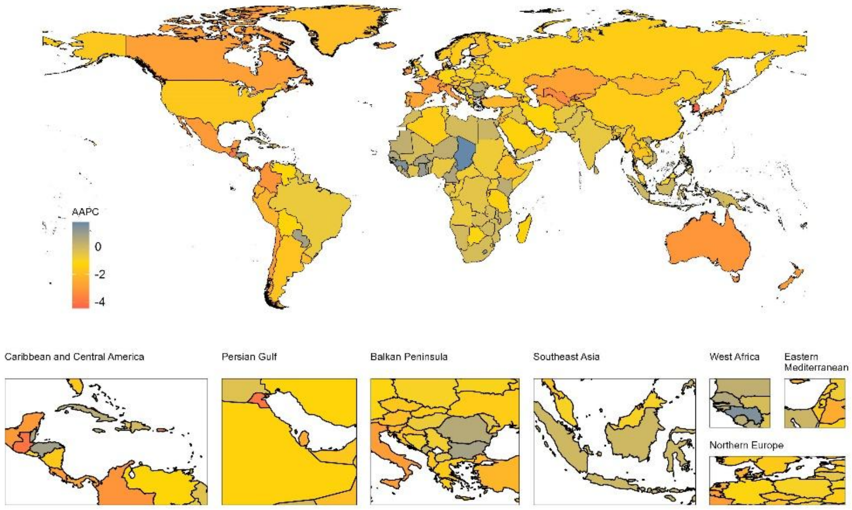
AAPC
0
-2
-4
Caribbean and Central America
Persian Gulf
Balkan Peninsula
Southeast Asia
West Africa
Eastern Mediterranean
Northern Europe

Figure S5. Global map of ASDR of laryngeal cancer in 1990(A) and 2021(B) and its AAPC(C).

Abbreviations: SDI: Sociodemographic Index, ASIR: age-standardised incidence rate, ASMR: age-standardised mortality rate, ASDR: age-standardised disability-adjusted life years rate.

Table S1. The incident cases and ASIR of larynx cancer among old adults in 1990 and 2021, along with the AAPC from 1990 to 2021, across 204 countries and territories.

204 countries and territories	1990		2021		1990-2021
	Incident cases (95 % UI)	ASIR per 100,000 (95 % UI)	Incident cases (95 % UI)	ASIR per 100,000 (95 % UI)	AAPC (95 % CI)
Afghanistan	146.76（78.15-234.01）	17.92（9.65-28.42）	130.09（72.25-200.18）	15.86（8.73-24.63）	-0.38 (-0.44 to -0.32)
Albania	47.64（34.6-63.9）	19.59（14.23-26.29）	90.95（61.12-130.42）	15.18（10.2-21.76）	-0.87 (-1.43 to -0.3)
Algeria	163.64（110.14-241.27）	12.51（8.4-18.46）	410.58（270.2-605.65）	10.42（6.84-15.37）	-0.59 (-0.7 to -0.49)
American Samoa	0.17（0.11-0.26）	7.79（5.12-11.45）	0.21（0.14-0.3）	3.99（2.69-5.7）	-2.04 (-2.63 to -1.45)
Andorra	1.19（0.7-1.97）	15.86（9.29-26.13）	2.02（1.13-3.23）	10.56（5.91-16.86）	-1.4 (-1.84 to -0.97)
Angola	41.88（25.13-64.25）	10.21（6.18-15.67）	105.54（69-156.6）	8.66（5.65-12.91）	-0.48 (-0.68 to -0.28)
Antigua and Barbuda	0.84（0.71-0.99）	12.1（10.19-14.2）	1.72（1.41-2.08）	12.88（10.55-15.56）	-0.37 (-2.16 to 1.44)
Argentina	855.33（696.38-1030.31）	20.32（16.55-24.49）	1005.03（805.41-1238.86）	13.97（11.2-17.23）	-1.09 (-1.37 to -0.81)
Armenia	79.86（72.32-87.76）	23.11（20.86-25.5）	74.71（63.55-87.32）	12.37（10.53-14.45）	-2.15 (-3.56 to -0.72)
Australia	255.72（213.53-307.14）	9.87（8.23-11.85）	326.96（259.47-403.95）	5.53（4.39-6.84）	-1.99 (-2.67 to -1.32)
Austria	221.69（181.91-269.47）	14.34（11.75-17.42）	229.1（180.44-286.47）	10.18（8.03-12.73）	-0.76 (-1.27 to -0.23)
Azerbaijan	87.02（70.95-105.17）	14.74（11.97-17.92）	142.31（103.81-202.2）	11.14（8.1-15.8）	-0.87 (-1.13 to -0.62)
Bahamas	2.69（2.29-3.13）	15.14（12.88-17.64）	7.61（5.92-9.76）	15.62（12.19-19.93）	0.26 (-0.12 to 0.64)
Bahrain	3.87（2.68-5.57）	23.77（16.38-34.2）	12.86（7.97-20.71）	15.8（10.03-24.56）	-1.26 (-1.87 to -0.65)
Bangladesh	1121.65（740.2-1636.16）	20.86（13.78-30.48）	2282.14（1438.74-3404.38）	13.64（8.59-20.37）	-1.19 (-1.47 to -0.91)
Barbados	3.84（3.31-4.43）	9.84（8.49-11.35）	8.24（6.2-10.56）	11.86（8.93-15.2）	0.63 (-0.38 to 1.65)
Belarus	309.81（250.38-379.37）	17.55（14.19-21.48）	384.44（276.68-517.97）	16.98（12.24-22.84）	-0.11 (-0.77 to 0.55)
Belgium	564.1（449.14-696.33）	27.84（22.17-34.36）	445.07（342.47-564.03）	15.17（11.71-19.22）	-2.05 (-2.4 to -1.69)
Belize	0.87（0.74-1）	7.61（6.49-8.79）	3.35（2.76-4.06）	9.88（8.13-11.96）	0.8 (-0.22 to 1.83)
Benin	13.05（8.57-18.92）	5.55（3.64-8.04）	34.66（22.29-51.53）	6.15（3.97-9.12）	0.35 (0.17 to 0.53)
Bermuda	1.44（1.14-1.79）	18.41（14.61-22.82）	2.8（2.08-3.74）	15.41（11.43-20.61）	-0.86 (-1.33 to -0.39)
Bhutan	3.48（2.08-5.52）	13.16（7.92-20.84）	8.56（5.34-12.98）	11.9（7.41-18.05）	-0.32 (-0.36 to -0.28)
Bolivia (Plurinational State of)	34.43（22.08-49.49）	9.5（6.11-13.67）	76.73（48.97-114.68）	7.15（4.58-10.65）	-0.92 (-0.99 to -0.85)
Bosnia and Herzegovina	113.08（91.7-136.81）	22.58（18.28-27.38）	178.5（126.8-234.76）	20.97（14.91-27.56）	-0.14 (-0.77 to 0.49)
Botswana	8.93（5.81-12.77）	13.23（8.64-18.89）	16.45（10.39-25.94）	9.82（6.27-15.26）	-0.94 (-1.12 to -0.75)
Brazil	1652.52（1524.93-1780.46）	15.68（14.41-16.91）	4577.48（4099.73-5039.08）	14.45（12.93-15.91）	-0.2 (-0.46 to 0.06)
Brunei Darussalam	1.95（1.36-2.74）	18.52（12.82-26.12）	3.15（2.19-4.45）	8.59（5.95-12.11）	-2.52 (-2.93 to -2.11)
Bulgaria	286.66（236.06-343.56）	16.8（13.79-20.2）	472.37（367.59-594.9）	25.15（19.56-31.69）	1.42 (0.38 to 2.47)
Burkina Faso	28.15（18.33-41.63）	5.49（3.58-8.12）	71.59（45.16-107.07）	6.92（4.38-10.37）	0.77 (0.64 to 0.91)
Burundi	30.26（18.45-45.24）	11.04（6.73-16.56）	39.92（25.6-60.52）	7.48（4.82-11.31）	-1.24 (-1.34 to -1.13)
Cabo Verde	2.89（1.84-4.31）	9.72（6.16-14.54）	5.72（3.34-8.62）	10.63（6.22-15.95）	0.31

					(-0.82 to 1.45)
Cambodia	52.86（34.79-76.69）	10.47（6.91-15.14）	144.71（91.09-228.36）	10.12（6.4-15.96）	-0.09<br>(-0.17 to -0.01)
Cameroon	29.43（19.44-43.07）	5.87（3.88-8.57）	102.07（60.96-160.62）	7.44（4.49-11.64）	0.76<br>(0.65 to 0.87)
Canada	858.42（697.64-1052.36）	20.17（16.39-24.72）	1001.96（790.29-1255.75）	10.34（8.16-12.96）	-2.01<br>(-2.8 to -1.22)
Central African Republic	14.22（8.06-21.66）	11.16（6.49-16.89）	19.6（11.21-30.33）	8.64（5.1-13.17）	-0.84<br>(-0.92 to -0.76)
Chad	15.51（10.08-22.91）	4.6（2.98-6.81）	49.97（30.4-76.37）	7.94（4.86-12.14）	1.82<br>(1.66 to 1.97)
Chile	125.76（103.36-152.65）	10.19（8.37-12.37）	196.9（154.9-245.61）	5.93（4.67-7.4）	-1.7<br>(-2.51 to -0.89)
China	9539.46（7699.93-11327.11）	9.86（7.98-11.67）	27244.29（21153.17-34725.59）	10.14（7.89-12.9）	0.06<br>(-0.1 to 0.22)
Colombia	324.76（273.38-379.98）	16.42（13.8-19.25）	531.73（398.95-689.44）	7.73（5.8-10.02）	-2.36<br>(-3.57 to -1.13)
Comoros	2.15（1.39-3.13）	9.4（6.09-13.68）	3.98（2.62-5.91）	7.2（4.72-10.66）	-0.9<br>(-1.02 to -0.77)
Congo	13.48（8.1-19.78）	10.98（6.69-16.08）	25.39（16.88-37.04）	9.24（6.14-13.59）	-0.55<br>(-0.71 to -0.39)
Cook Islands	0.06（0.04-0.08）	4.3（2.87-6.05）	0.11（0.08-0.17）	3.36（2.22-5.16）	-0.8<br>(-1.22 to -0.38)
Costa Rica	27.04（22-32.9）	13.05（10.61-15.88）	43.16（33.15-55.6）	6.26（4.81-8.06）	-2.46<br>(-3.1 to -1.83)
Coted'Ivoire	30.09（19.97-43.46）	7.08（4.72-10.23）	76.94（47.3-124.41）	6.42（3.99-10.24）	-0.32<br>(-0.43 to -0.21)
Croatia	218.21（178.71-264.34）	27.19（22.28-32.97）	230.71（178.22-294.01）	19.65（15.14-25.09）	-0.95<br>(-1.5 to -0.39)
Cuba	409.52（340.16-490.59）	31.83（26.42-38.13）	1025.49（790.33-1312.33）	42.35（32.61-54.24）	0.94<br>(-0.02 to 1.92)
Cyprus	13.77（9.28-19.92）	14.2（9.48-20.83）	31.4（20.94-45.47）	11.47（7.64-16.61）	-0.79<br>(-1.13 to -0.46)
Czechia	279.39（230.47-337.27）	15（12.36-18.12）	346.03（272.42-434.26）	12.66（9.96-15.89）	-0.51<br>(-0.9 to -0.12)
Democratic People's Republic of Korea	111.41（70.75-162.79）	6.1（3.9-8.86）	249.76（161.72-360.12）	6.23（4.04-8.98）	0.05<br>(-0.05 to 0.15)
Democratic Republic of the Congo	144.05（89.1-215.1）	8.48（5.3-12.67）	286.04（179.69-424.98）	7.34（4.64-10.93）	-0.48<br>(-0.66 to -0.3)
Denmark	209.64（171.55-254.66）	20.14（16.49-24.44）	214.09（168.19-267.51）	13.92（10.95-17.37）	-1.15<br>(-1.86 to -0.42)
Djibouti	1.42（0.88-2.23）	10.34（6.48-16.15）	6.34（3.87-9.72）	9.56（5.9-14.58）	-0.25<br>(-0.33 to -0.17)
Dominica	1.14（0.83-1.51）	14.5（10.6-19.28）	1.67（1.14-2.35）	15.55（10.62-21.85）	0.2<br>(0.1 to 0.31)
Dominican Republic	40.95（29.44-56.14）	9.81（7.04-13.5）	120.18（78.01-176.84）	9.95（6.46-14.64）	0.04<br>(-0.35 to 0.44)
Ecuador	39.43（32.65-47.1）	6.59（5.45-7.88）	81.21（58.49-109.85）	4.07（2.93-5.49）	-1.68<br>(-3.03 to -0.31)
Egypt	188.73（138.5-263.49）	6.76（4.93-9.43）	582.75（407.54-827.49）	8.56（6.01-12.12）	0.77<br>(0.44 to 1.11)
El Salvador	20.52（16.05-25.64）	5.86（4.58-7.33）	38.09（27.83-51.57）	4.92（3.59-6.66）	-0.4<br>(-0.93 to 0.13)
Equatorial Guinea	2.16（1.23-3.4）	9.91（5.73-15.51）	3.97（2.25-6.46）	7.7（4.42-12.49）	-0.8<br>(-1.01 to -0.6)
Eritrea	10.56（6.67-15.66）	9.34（5.97-13.78）	21.66（14.43-31.29）	7.59（5.08-10.96）	-0.67<br>(-0.77 to -0.58)
Estonia	48.35（38.95-58.9）	17.93（14.45-21.85）	54.97（41.22-71.92）	16.34（12.23-21.41）	-0.33<br>(-1.72 to 1.09)
Eswatini	4.64（2.75-6.83）	14.48（8.64-21.27）	8.43（4.82-12.55）	12.76（7.34-18.96）	-0.4<br>(-0.6 to -0.2)
Ethiopia	186.12（108.83-273.67）	8.13（4.83-11.91）	247.46（171.56-348.11）	5.2（3.61-7.31）	-1.45<br>(-1.54 to -1.35)
Fiji	1.67（1.14-2.41）	4.7（3.2-6.88）	3.98（2.56-5.94）	4.69（3.01-7.01）	0.01<br>(-0.47 to 0.48)
Finland	90.33（73.43-110.88）	9.63（7.82-11.82）	130.25（99.99-166.56）	7.97（6.14-10.18）	-0.59<br>(-1.26 to 0.08)

France	3760.58（3032.31-4636.87）	35.59（28.68-43.87）	3889.95（2979.39-4966.34）	22.64（17.37-28.94）	-1.39 (-1.78 to -0.99)
Gabon	7.78（4.93-11.69）	11.12（7.05-16.76）	11.75（7.52-17.18）	9.83（6.34-14.32）	-0.37 (-0.5 to -0.23)
Gambia	1.07（0.72-1.54）	2.71（1.81-3.89）	2.9（1.9-4.21）	2.66（1.74-3.86）	-0.09 (-0.55 to 0.38)
Georgia	182.48（160.5-206.8）	21.75（19.11-24.64）	164.83（139.83-192.43）	20.47（17.37-23.88）	-0.05 (-2.24 to 2.18)
Germany	1922.65（1571.82-2331.41）	11.91（9.74-14.44）	2959.65（2310.41-3716.61）	12.39（9.7-15.53）	0.18 (-0.24 to 0.6)
Ghana	31.51（20.83-46.84）	4.6（3.04-6.85）	125.96（76.3-186.52）	6.78（4.14-10.02）	1.26 (1.12 to 1.4)
Greece	678.4（573.89-796.46）	33.73（28.52-39.6）	700.32（575.32-838.08）	23.75（19.55-28.46）	-1.18 (-1.43 to -0.92)
Greenland	0.57（0.41-0.79）	15.52（10.99-21.52）	0.88（0.6-1.29）	9.92（6.7-14.48）	-1.22 (-1.6 to -0.85)
Grenada	0.87（0.7-1.07）	9.23（7.46-11.38）	1.1（0.85-1.38）	7.71（5.95-9.72）	-0.44 (-1.84 to 0.99)
Guam	0.36（0.27-0.48）	4.61（3.36-6.01）	0.51（0.38-0.67）	1.9（1.42-2.51）	-2.65 (-3.83 to -1.45)
Guatemala	36.19（32.14-40.43）	10.14（9.02-11.32）	41.78（34.05-50.61）	3.22（2.63-3.89）	-3.73 (-4.68 to -2.77)
Guinea	19.01（12.22-27.66）	4.72（3.03-6.88）	47.26（29.48-71.9）	7.37（4.62-11.18）	1.47 (1.38 to 1.57)
Guinea-Bissau	3.56（2.01-5.52）	7.74（4.45-11.91）	6.68（3.96-9.87）	8.64（5.2-12.73）	0.36 (0.29 to 0.44)
Guyana	2.99（2.37-3.79）	6.78（5.37-8.59）	4.74（3.3-6.53）	6.04（4.23-8.3）	-0.31 (-1.27 to 0.66)
Haiti	56.54（33.73-85.13）	15.22（9.15-22.9）	101.17（59.85-156.12）	13.12（7.78-20.32）	-0.46 (-0.54 to -0.38)
Honduras	16（11.33-21.96）	7（4.94-9.63）	70.23（45.58-100.17）	9.65（6.25-13.76）	1.12 (0.86 to 1.38)
Hungary	439.92（362.05-531.56）	22.04（18.15-26.65）	574.71（446.98-727.05）	23.08（17.88-29.29）	0.18 (-0.47 to 0.84)
Iceland	3.79（3.04-4.72）	10.3（8.24-12.82）	4.69（3.62-5.94）	6.16（4.76-7.81）	-1.56 (-2.08 to -1.05)
India	8059.95（6431.58-9883.29）	15.37（12.18-18.91）	20960.34（17790.35-24562.9）	14.37（12.19-16.84）	-0.21 (-0.63 to 0.22)
Indonesia	686.21（488.83-866.21）	6.44（4.56-8.18）	1970.7（1312.88-2624.69）	7.28（4.83-9.72）	0.4 (0.33 to 0.47)
Iran (Islamic Republic of)	525.76（432.58-617.73）	18.02（14.59-21.24）	1559.27（1294.97-1850.32）	17.49（14.48-20.74）	-0.16 (-0.34 to 0.03)
Iraq	139.57（93.46-198.99）	15.44（10.35-22.01）	472.22（301.32-686.97）	18.17（11.65-26.42）	0.54 (0.3 to 0.77)
Ireland	100.1（80.88-122.21）	18.48（14.92-22.58）	110.26（85.58-140.03）	10.74（8.35-13.64）	-1.74 (-2.74 to -0.73)
Israel	69.41（55.83-85.54）	10.95（8.79-13.5）	147.75（113.96-186.98）	9.24（7.14-11.68）	-0.48 (-1.38 to 0.43)
Italy	3548.01（3127.53-4039.47）	29.94（26.35-34.11）	3000.09（2547.41-3484.31）	16.73（14.28-19.46）	-1.91 (-2.45 to -1.36)
Jamaica	19.03（15.13-23.55）	8.21（6.53-10.16）	37.54（25.66-52.48）	9.78（6.69-13.67）	0.74 (0.34 to 1.14)
Japan	2370.2（2111.71-2632.7）	10.89（9.69-12.09）	3396.31（2881.21-3851.27）	6.88（5.93-7.71）	-1.4 (-2.09 to -0.71)
Jordan	14.15（9.38-20.43）	10.29（6.85-14.89）	53.21（34.02-81.24）	6.76（4.34-10.28）	-1.3 (-1.68 to -0.92)
Kazakhstan	257.23（228.45-288.3）	15.91（14.1-17.85）	181.68（153.91-211.71）	7.68（6.51-8.95）	-2.21 (-2.83 to -1.58)
Kenya	58.94（42.1-79.98）	6.31（4.51-8.56）	188.84（139.01-240.46）	7.23（5.33-9.2）	0.44 (0.33 to 0.56)
Kiribati	0.05（0.03-0.07）	1.19（0.78-1.72）	0.1（0.06-0.15）	1.26（0.78-1.93）	0.17 (0.08 to 0.26)
Kuwait	7.82（6.12-9.83）	13.33（10.42-16.78）	14.5（10.29-19.73）	5.44（3.88-7.39）	-2.77 (-6.29 to 0.89)
Kyrgyzstan	37.37（29.43-46.99）	9.69（7.64-12.17）	28.46（20.95-37.08）	4.78（3.53-6.22）	-2.37 (-3.54 to -1.19)

Lao People's Democratic Republic	24.07（15.23-36.24）	9.9（6.3-14.86）	37.75（23.46-58.5）	7.47（4.66-11.56）	-0.9<br>(-0.97 to -0.84)
Latvia	77.21（64.31-93.02）	16.4（13.65-19.76）	63.64（49.31-80.79）	12.62（9.77-16.03）	-0.77<br>(-1.79 to 0.26)
Lebanon	53.74（33.35-79.72）	20.78（13.04-30.68）	153.54（104.13-220.26）	20.39（13.83-29.25）	-0.02<br>(-0.32 to 0.29)
Lesotho	11.29（7.44-16.37）	10.97（7.23-15.88）	20.66（13.06-30.28）	15.16（9.61-22.16）	1.12<br>(0.84 to 1.41)
Liberia	7.28（4.59-11.43）	5.29（3.33-8.31）	13.55（8.3-21.33）	6.38（3.92-10）	0.62<br>(0.36 to 0.87)
Libya	46.08（29.92-69.76）	21.6（14.04-32.62）	136.39（87.57-207.01）	25.01（16.08-37.87）	0.54<br>(0.04 to 1.05)
Lithuania	105.2（86.31-127.24）	17.99（14.76-21.78）	120.57（92.42-153.05）	16.86（12.91-21.41）	0<br>(-1.37 to 1.39)
Luxembourg	17.38（14.73-20.43）	24.46（20.72-28.76）	19.84（16.08-24.24）	15.02（12.18-18.38）	-1.64<br>(-2.65 to -0.61)
Madagascar	47.86（31.51-67.77）	8.16（5.38-11.58）	65.69（42.71-96.57）	5.62（3.68-8.23）	-1.19<br>(-1.25 to -1.12)
Malawi	13.39（9.15-18.96）	3（2.05-4.25）	25.09（17.04-37.21）	3（2.04-4.43）	0.03<br>(-0.11 to 0.17)
Malaysia	113.94（74.69-164.64）	10.99（7.19-15.92）	368.35（247.15-524.22）	10.66（7.15-15.17）	-0.21<br>(-0.49 to 0.07)
Maldives	0.73（0.46-1.09）	8.26（5.26-12.53）	1.54（1-2.26）	4.59（2.99-6.7）	-2.02<br>(-2.49 to -1.55)
Mali	26.09（18.47-36.15）	5.73（4.05-7.97）	53.79（35.19-79.81）	5.51（3.61-8.16）	-0.08<br>(-0.28 to 0.12)
Malta	11.36（8.94-14.29）	20.52（16.14-25.83）	13.74（10.48-17.66）	10.57（8.07-13.6）	-2.21<br>(-2.42 to -2)
Marshall Islands	0.09（0.05-0.14）	4.84（2.9-7.54）	0.16（0.09-0.25）	4.89（2.85-7.7）	0.08<br>(-0.16 to 0.32)
Mauritania	5.99（3.94-8.71）	5.18（3.39-7.54）	15.98（9.72-25.07）	6.45（3.94-10.09）	0.75<br>(0.53 to 0.97)
Mauritius	13.15（11.23-15.41）	14.61（12.48-17.09）	29.66（25.04-34.69）	12.2（10.31-14.25）	-0.73<br>(-2.69 to 1.28)
Mexico	635.74（606.56-662.69）	13.67（13-14.26）	908.96（773.4-1057.32）	6（5.11-6.97）	-2.62<br>(-3 to -2.23)
Micronesia (Federated States of)	0.28（0.18-0.42）	4.84（3.07-7.47）	0.34（0.21-0.52）	4.25（2.68-6.51）	-0.43<br>(-0.52 to -0.34)
Monaco	6.01（3.87-8.8）	66.36（42.62-97.53）	5.92（3.96-8.57）	47.87（31.96-69.41）	-1.05<br>(-1.21 to -0.89)
Mongolia	9.32（6.46-12.76）	7.49（5.19-10.27）	11.19（7.75-15.78）	4.39（3.05-6.21）	-1.76<br>(-2.41 to -1.11)
Montenegro	26.86（20.77-34.7）	33.98（26.27-43.89）	53.13（40.49-70.3）	39.4（30.01-52.02）	0.64<br>(0.35 to 0.94)
Morocco	200.71（133.86-289.33）	11.86（7.9-17.12）	576.11（360.02-870.23）	13.75（8.59-20.74）	0.49<br>(0.34 to 0.64)
Mozambique	64.52（41.18-93.77）	9.61（6.17-13.96）	113.39（74.82-163.03）	9.23（6.11-13.26）	-0.1<br>(-0.22 to 0.01)
Myanmar	255.3（155.62-381.83）	9.45（5.83-14.05）	351.68（222.9-527.62）	6.15（3.92-9.21）	-1.38<br>(-1.43 to -1.34)
Namibia	10.57（7.4-14.72）	13.22（9.25-18.4）	21.69（14.44-31.16）	13.61（9.11-19.51）	0.12<br>(-0.02 to 0.25)
Nauru	0.03（0.02-0.04）	6.71（4.09-10.06）	0.03（0.02-0.04）	4.81（2.92-7.37）	-1.08<br>(-1.16 to -1)
Nepal	165.31（103.18-249.25）	15.83（9.86-23.9）	374.95（237.7-564.49）	13.33（8.44-20.09）	-0.53<br>(-0.62 to -0.43)
Netherlands	479.57（389.86-589.96）	18.56（15.09-22.83）	567.38（444.97-712.43）	12.14（9.53-15.22）	-1.38<br>(-1.58 to -1.18)
New Zealand	80.86（63.85-101.78）	15.54（12.26-19.57）	98.1（75.28-124.79）	8.71（6.69-11.07）	-1.73<br>(-2.15 to -1.3)
Nicaragua	13.31（9.56-18.1）	7.87（5.65-10.7）	32（22.12-46.65）	5.62（3.88-8.17）	-1.19<br>(-1.41 to -0.97)
Niger	15.45（9.56-23.22）	4.97（3.11-7.43）	51.94（30.5-81.75）	5.81（3.46-9.07）	0.51<br>(0.33 to 0.69)
Nigeria	379.46（262.49-514.09）	7.29（5.1-9.81）	623.41（436.1-854）	6.58（4.66-8.93）	-0.33<br>(-0.39 to -0.27)
Niue	0.01（0.01-0.02）	3.66（2.35-5.48）	0.01（0.01-0.02）	3.83（2.48-5.73）	0.14<br>(0.09 to 0.18)

North Macedonia	53.97（41.67-69.01）	23.22（17.92-29.84）	108.28（77.97-145.69）	23.97（17.35-32.09）	0.11 (-0.13 to 0.36)
Northern Mariana Islands	0.11（0.07-0.17）	8.86（5.6-13.44）	0.31（0.21-0.45）	5.74（3.79-8.31）	-1.42 (-1.86 to -0.97)
Norway	89.67（77.8-102.97）	9.93（8.61-11.41）	107.08（88.96-126.48）	8.14（6.77-9.62）	-0.55 (-1.76 to 0.68)
Oman	4.11（2.61-6.2）	5.69（3.62-8.57）	9.19（5.84-13.96）	4.8（3.02-7.27）	-0.56 (-1.28 to 0.15)
Pakistan	1831.17（1377.23-2419.51）	27.79（20.81-36.8）	3424.64（2415.44-4717.33）	25.95（18.32-35.64）	-0.22 (-0.32 to -0.12)
Palau	0.05（0.03-0.07）	3.99（2.53-5.95）	0.08（0.05-0.12）	3.27（2.01-5.05）	-0.71 (-0.88 to -0.54)
Palestine	10.02（6.57-14.94）	10.06（6.61-14.96）	22.45（15.34-32.02）	8.35（5.7-11.91）	-0.61 (-0.84 to -0.38)
Panama	20.64（17.82-23.78）	11.8（10.17-13.58）	33.74（25.01-42.7）	6.15（4.56-7.79）	-2.07 (-2.43 to -1.71)
Papua New Guinea	5.97（3.54-9.45）	3.44（2.05-5.49）	15.06（9.29-23.93）	3.26（1.99-5.23）	-0.14 (-0.27 to -0.02)
Paraguay	20.73（14.49-28.6）	7.85（5.48-10.83）	79.41（51.48-117.36）	11.17（7.26-16.48）	1.16 (0.67 to 1.66)
Peru	101.88（72.68-136.19）	7.51（5.36-10.05）	190.17（125.23-280.61）	4.7（3.09-6.93）	-1.39 (-2.27 to -0.5)
Philippines	183.55（149.67-232.14）	5.81（4.75-7.32）	555.23（444.78-674.13）	5.79（4.66-7.02）	0.04 (-0.07 to 0.15)
Poland	1189.76（1119.23-1264.08）	20.35（19.13-21.62）	1971.51（1724.24-2225.24）	20.06（17.54-22.64）	-0.08 (-0.43 to 0.27)
Portugal	351.87（290.85-425.76）	18.84（15.57-22.79）	359.49（283.66-447.15）	11.73（9.28-14.58）	-1.59 (-2.06 to -1.12)
Puerto Rico	80.33（64.76-97.61）	17.24（13.9-20.96）	75.85（56.21-100.14）	8.13（6.02-10.75）	-2.46 (-3.88 to -1.01)
Qatar	1.81（1.19-2.71）	23.57（15.56-35.37）	12（7.26-19.14）	18.47（11.28-29.31）	-0.76 (-1.7 to 0.19)
Republic of Korea	741.32（482.82-1000.43）	22.21（14.46-29.94）	1636.91（1112.21-2290.95）	13.27（9.02-18.58）	-1.6 (-2.1 to -1.1)
Republic of Moldova	101.87（88.74-115.95）	17.5（15.25-19.91）	141.44（117.49-168.45）	17.02（14.15-20.26）	-0.02 (-1.38 to 1.35)
Romania	557.31（454.93-672.51）	14.88（12.13-17.96）	1141.53（881.3-1454.45）	23.54（18.14-30.03）	1.61 (1.03 to 2.19)
Russian Federation	4162.61（4000.25-4318.47）	16.73（16.05-17.37）	4553.89（4018.4-5023.14）	13.5（11.92-14.88）	-0.56 (-1.3 to 0.18)
Rwanda	43.62（27.98-64.85）	13.06（8.4-19.45）	59.24（37.08-91.73）	8.18（5.16-12.62）	-1.5 (-1.61 to -1.38)
Saint Kitts and Nevis	0.49（0.41-0.57）	8.94（7.62-10.45）	0.99（0.77-1.25）	11.22（8.79-14.05）	0.85 (-0.4 to 2.12)
Saint Lucia	1.65（1.43-1.9）	14.89（12.88-17.07）	4.25（3.28-5.34）	14.21（10.95-17.81）	-0.08 (-0.95 to 0.8)
Saint Vincent and the Grenadines	1.37（1.17-1.59）	14.66（12.54-16.98）	3.11（2.59-3.72）	16.98（14.15-20.24）	0.34 (-0.58 to 1.28)
Samoa	0.18（0.12-0.26）	1.77（1.22-2.54）	0.28（0.19-0.41）	1.63（1.1-2.42）	-0.24 (-0.36 to -0.12)
San Marino	1.34（0.88-1.95）	29.17（19.27-42.62）	1.2（0.65-1.97）	13.32（7.12-21.97）	-2.66 (-2.81 to -2.51)
Sao Tome and Principe	0.35（0.22-0.51）	4.32（2.77-6.3）	0.66（0.43-0.98）	5.49（3.54-8.16）	0.75 (0.39 to 1.1)
Saudi Arabia	35.24（22.9-51.47）	5.78（3.76-8.44）	101.39（67.83-148.6）	5.89（3.94-8.62）	0.06 (-0.04 to 0.16)
Senegal	21.76（14.39-31.08）	5.75（3.8-8.23）	62.79（37.81-95.28）	7.1（4.28-10.79）	0.73 (0.37 to 1.09)
Serbia	366.16（253.55-522.1）	24.63（17.09-35.16）	565.6（387.12-790.77）	25.27（17.28-35.32）	0.2 (0.02 to 0.39)
Seychelles	2.41（1.65-3.35）	33.18（22.74-46.09）	4.25（2.86-6.04）	30.69（20.73-43.54）	-0.28 (-0.71 to 0.16)
Sierra Leone	13.67（8.92-20.07）	5.6（3.65-8.24）	26.97（17.02-41.07）	6.47（4.1-9.83）	0.47 (0.4 to 0.54)
Singapore	51.86（41.29-64.95）	19.99（15.91-24.99）	113.58（86.17-146.51）	10.36（7.86-13.37）	-2.12 (-3.99 to -0.2)

Slovakia	167.05（119.03-228.83）	20.77（14.79-28.44）	208.11（141.11-300.93）	16.05（10.88-23.21）	-0.85 (-0.98 to -0.73)
Slovenia	56.1（45.94-67.8）	17.61（14.42-21.31）	88.95（67.72-114.48）	15.75（11.99-20.28）	-0.66 (-1.61 to 0.31)
Solomon Islands	0.67（0.39-1.05）	5（3.02-7.81）	1.4（0.87-2.15）	4.2（2.61-6.43）	-0.58 (-0.72 to -0.44)
Somalia	23.77（14.07-36.85）	10.44（6.24-16.11）	53.3（32.04-80.73）	7.98（4.86-12.02）	-0.85 (-0.94 to -0.76)
South Africa	221.27（170.56-320.41）	9.09（7.02-13.12）	513.64（445.63-583.57）	9.05（7.84-10.3）	-0.06 (-0.43 to 0.32)
South Sudan	33.19（20.34-50.95）	10.51（6.45-16.13）	33.16（19.59-51.56）	8.37（4.96-12.99）	-0.72 (-0.8 to -0.64)
Spain	2501.42（2028.98-3046.4）	34.17（27.71-41.62）	2601.56（1971.59-3350.59）	22.26（16.87-28.69）	-1.39 (-1.94 to -0.84)
Sri Lanka	73.08（51.12-103.88）	6.14（4.29-8.75）	305.44（168.52-489.27）	8.58（4.78-13.7）	1.06 (0.51 to 1.62)
Sudan	146.09（83.41-257.83）	13.69（7.77-24.45）	260.88（157.81-398.95）	12.76（7.72-19.54）	-0.23 (-0.28 to -0.17)
Suriname	1.55（1.13-2.1）	5.25（3.81-7.08）	3.7（2.31-5.55）	4.75（2.97-7.13）	-0.23 (-1 to 0.54)
Sweden	154.36（125.92-188.86）	7.74（6.32-9.47）	172.21（132.73-217.57）	6.14（4.73-7.76）	-0.6 (-2.06 to 0.88)
Switzerland	221.55（177.22-274.33）	17.03（13.61-21.09）	221.48（168.58-285.17）	9.82（7.48-12.66）	-1.88 (-2.32 to -1.44)
Syrian Arab Republic	54.07（38.55-76.35）	9.47（6.75-13.41）	155.65（104.54-217.59）	10.5（7.13-14.57）	0.29 (-0.05 to 0.63)
Taiwan (Province of China)	227.25（185.22-278.14）	11.16（9.08-13.67）	472.39（358.79-610.7）	8.33（6.34-10.77）	-0.84 (-1.31 to -0.37)
Tajikistan	26.01（19.1-34.5）	7.9（5.82-10.47）	31.49（20.75-44.49）	4.83（3.1-6.85）	-1.72 (-2.02 to -1.43)
Thailand	535.17（383.9-743.55）	13.8（9.86-19.18）	1549.52（1005.18-2305.82）	10.98（7.14-16.33）	-0.74 (-1.02 to -0.45)
Timor-Leste	1.58（0.99-2.43）	6.17（3.9-9.41）	6.39（4.04-9.65）	6.06（3.83-9.19）	-0.05 (-0.19 to 0.1)
Togo	7.86（5.14-11.61）	5.87（3.84-8.65）	30.28（18.55-45.76）	7.3（4.52-10.99）	0.7 (0.48 to 0.92)
Tokelau	0.01（0-0.01）	4.23（2.61-6.66）	0.01（0-0.01）	3.44（2.15-5.38）	-0.67 (-0.73 to -0.6)
Tonga	0.25（0.15-0.39）	3.97（2.46-6.29）	0.36（0.22-0.56）	3.79（2.32-5.89）	-0.17 (-0.53 to 0.19)
Trinidad and Tobago	9.35（8.11-10.78）	9.03（7.84-10.39）	19.99（14.68-26.27）	7.74（5.7-10.15）	-0.52 (-0.65 to -0.4)
Tunisia	116.25（76.81-169.28）	19.48（12.87-28.31）	341.93（203.88-539.2）	20.38（12.18-32.04）	0.14 (-0.03 to 0.32)
Turkey	918.65（593.27-1399.63）	23.24（15.03-35.38）	2212.89（1478.57-3208.51）	18.89（12.63-27.36）	-0.67 (-1.06 to -0.28)
Turkmenistan	28.99（25.59-32.54）	12.47（10.97-14.05）	28.37（21.56-36.86）	5.84（4.45-7.56）	-2.39 (-2.89 to -1.89)
Tuvalu	0.03（0.02-0.05）	4.1（2.71-5.95）	0.05（0.03-0.07）	3.81（2.47-5.58）	-0.24 (-0.3 to -0.18)
Uganda	87.85（59.65-123.73）	11.84（8.04-16.65）	151.41（98.48-228.2）	9.58（6.26-14.35）	-0.71 (-0.81 to -0.61)
Ukraine	1597.37（1302.88-1921.96）	15.98（13.04-19.23）	1177.3（755.99-1733.91）	11.01（7.08-16.2）	-1.24 (-1.67 to -0.82)
United Arab Emirates	4.05（2.29-6.38）	11.91（6.86-18.64）	23.37（15.08-34.59）	10.02（6.61-14.55）	-0.73 (-2.16 to 0.73)
United Kingdom	1984.67（1887.77-2072.58）	16.61（15.8-17.34）	2349.49（2160.26-2501.17）	14.01（12.95-14.88）	-0.59 (-0.92 to -0.27)
United Republic of Tanzania	126.83（82.38-184.72）	9.87（6.44-14.37）	192.9（122.45-305.52）	6.85（4.37-10.76）	-1.17 (-1.27 to -1.06)
United States of America	1.09（0.76-1.55）	20.75（19.65-21.64）	1.86（1.17-2.79）	15.81（14.63-16.7）	-1.45 (-1.96 to -0.95)
United States Virgin Islands	8667.27（8200.05-9040.58）	11.16（7.76-15.85）	12421.01（11488.72-13128.84）	7.35（4.61-11.03）	-0.95 (-1.15 to -0.75)
Uruguay	174.1（141.11-213.13）	33.58（27.22-41.12）	156.02（121.71-195.56）	22.32（17.41-28）	-1.38 (-1.52 to -1.24)

Uzbekistan	147.2（121.76-175.7）	10.53（8.73-12.55）	120.43（92.11-154.92）	3.94（3.02-5.05）	-3.23 (-3.99 to -2.46)
Vanuatu	0.23（0.14-0.37）	3.75（2.25-5.89）	0.54（0.34-0.83）	3.07（1.92-4.71）	-0.65 (-0.8 to -0.49)
Venezuela (Bolivarian Republic of)	193.62（171.2-216.78）	17.64（15.56-19.76）	539.66（397.34-722.37）	14.37（10.62-19.17）	-0.78 (-1.15 to -0.41)
Viet Nam	388.98（260.01-557.86）	7.81（5.24-11.22）	1358.29（857.81-2052.2）	11.16（7.12-16.78）	1.17 (1.05 to 1.3)
Yemen	82.12（48.54-129.35）	14.79（8.7-23.41）	234.57（135.25-365.89）	15.53（8.95-24.35）	0.16 (0.02 to 0.3)
Zambia	34.76（22.84-50.4）	11.14（7.33-16.17）	77.66（38.57-169.03）	10.61（5.39-22.57）	-0.14 (-0.34 to 0.07)
Zimbabwe	56.26（38.41-79.43）	11.67（7.99-16.44）	84.95（57.77-121.39）	10.83（7.39-15.45）	-0.22 (-0.39 to -0.04)

Abbreviations: SDI: Sociodemographic Index; ASIR: age-standardised incidence rate; AAPC=average annual percentage change; CI = confidence interval; UI = uncertainty interval.

Table S2. The death cases and ASMR of older adults with larynx cancer in 1990 and 2021, and its AAPC from 1990 to 2021

Characteristics	1990		2021		1990-2021
	Death cases (95 % UI)	ASMR (95 % UI)	Death cases (95 % UI)	ASMR (95 % UI)	AAPC (95 % CI)
Global	54179.57（50351.82-57912.91）	11.31（10.48-12.1）	82892.77（76126.84-89824.56）	7.66（7.03-8.31）	-1.23 (-1.3 to -1.17)
High SDI	12390.62（11708.24-13020.68）	8.57（8.09-9）	13004.04（11842.1-13851.81）	4.6（4.22-4.89）	-2.02 (-2.11 to -1.93)

High-middle SDI	16813.62（15756.71-17944.12）	13.47（12.58-14.41）	19350.29（17282.11-21479.74）	7.58（6.76-8.41）	-1.83<br>(-2.07 to -1.6)
Low SDI	3061.52（2414.96-3824.87）	12.24（9.63-15.3）	5640.55（4758.55-6620.81）	10.38（8.74-12.18）	-0.48<br>(-0.62 to -0.33)
Low-middle SDI	9409.33（7859.26-11189.68）	13.88（11.55-16.53）	19558.86（17220.21-22505.8）	11.72（10.31-13.5）	-0.52<br>(-0.86 to -0.18)
Middle SDI	12420.39（11151.58-13540.05）	10.98（9.84-11.96）	25224.22（22421.2-28291.79）	7.87（6.98-8.82）	-1.07<br>(-1.19 to -0.95)
Region					
Andean Latin America	175.46（142.65-215.08）	7.68（6.23-9.43）	292.59（217.59-381.99）	4.13（3.07-5.39）	-1.98<br>(-2.53 to -1.42)
Australasia	216.26（184.25-254.16）	6.98（5.94-8.19）	227.41（183.6-275.51）	3.1（2.51-3.75）	-2.77<br>(-3.35 to -2.19)
Caribbean	553.44（486.34-633.21）	17.54（15.39-20.08）	1069.98（877.86-1292.5）	15.9（13.05-19.2）	-0.3<br>(-1 to 0.4)
Central Asia	764.87（716.37-814.63）	13.29（12.42-14.17）	636.53（566.69-713.93）	6.56（5.83-7.35）	-2.42<br>(-2.59 to -2.24)
Central Europe	3247.21（3042.28-3470.54）	16.46（15.4-17.61）	4024.09（3645.04-4419.32）	13.39（12.13-14.71）	-0.65<br>(-0.85 to -0.46)
Central Latin America	1219.85（1146.17-1287.61）	13.48（12.61-14.24）	1829.17（1586.95-2094.15）	6.09（5.28-6.96）	-2.62<br>(-3.1 to -2.14)
Central Sub-Saharan Africa	223.48（150.22-313.31）	9.52（6.45-13.36）	432（305.1-588.83）	7.8（5.49-10.74）	-0.64<br>(-0.79 to -0.49)
East Asia	8731.14（7088.56-10282.6）	9.1（7.42-10.69）	15575.8（12226-19543.7）	5.78（4.55-7.22）	-1.47<br>(-1.76 to -1.18)
Eastern Europe	5060.4（4777.88-5350.41）	13.36（12.6-14.14）	4147.65（3664.54-4661.29）	8.51（7.52-9.56）	-1.41<br>(-1.98 to -0.84)
Eastern Sub-Saharan Africa	766.12（601.24-941.85）	9.24（7.26-11.34）	1226.89（977.43-1538.02）	6.85（5.45-8.54）	-0.95<br>(-1.01 to -0.9)
High-income Asia Pacific	1288.9（1119.53-1444.03）	5.26（4.56-5.88）	1527.75（1282.09-1731.58）	2.18（1.85-2.48）	-2.79<br>(-3.15 to -2.44)
High-income North America	3623.91（3422.8-3773.47）	7.75（7.32-8.07）	4188.45（3840.79-4430.62）	4.69（4.31-4.96）	-1.66<br>(-1.83 to -1.49)
North Africa and Middle East	2520.77（2046.7-3122.63）	13.9（11.21-17.36）	4766.31（4020.38-5570.82）	9.89（8.31-11.54）	-1.1<br>(-1.15 to -1.04)
Oceania	10.09（7.21-14.08）	3.69（2.65-5.16）	22.56（16.19-31.7）	3.28（2.34-4.64）	-0.34<br>(-0.42 to -0.27)
South Asia	10943.11（8971.26-13138.54）	17.37（14.16-20.91）	24028.33（20703.12-27848.13）	13.8（11.88-16）	-0.68<br>(-0.96 to -0.4)
Southeast Asia	2087.61（1751.03-2458.35）	7.57（6.33-8.93）	4823.46（4030.15-5805.38）	6.47（5.39-7.81）	-0.51<br>(-0.59 to -0.43)
Southern Latin America	938.86（804.32-1088.41）	15.94（13.65-18.49）	937.76（785.2-1101.89）	8.28（6.94-9.73）	-2.04<br>(-2.27 to -1.81)
Southern Sub-Saharan Africa	292.25（234.98-389.01）	9.39（7.56-12.48）	580.6（506.29-658.43）	8.57（7.46-9.72）	-0.28<br>(-0.55 to -0.01)
Tropical Latin America	1516（1398.42-1630.79）	14.41（13.22-15.53）	3667.53（3297.82-4014.98）	11.46（10.29-12.55）	-0.68<br>(-0.98 to -0.38)
Western Europe	9348.55（8724.29-9949.56）	12.2（11.38-12.98）	7540.71（6669.9-8272.73）	6.01（5.36-6.58）	-2.28<br>(-2.42 to -2.14)
Western Sub-Saharan Africa	651.29（517.68-810.56）	6.55（5.24-8.1）	1347.22（1082.58-1627.11）	6.65（5.37-7.99）	0.05<br>(-0.01 to 0.11)
204 countries and territories					
Afghanistan	147.57（77.78-234.6）	18.66（9.98-29.58）	127.5（70.27-195.39）	15.7（8.58-24.29）	-0.55<br>(-0.61 to -0.48)
Albania	43.94（32.18-59）	18.51（13.55-24.88）	66.89（45.47-95.77）	11.35（7.72-16.25）	-1.59<br>(-2.02 to -1.16)
Algeria	144.45（97.88-212.67）	11.88（8.01-17.57）	271.43（180.68-395.09）	7.42（4.92-10.84）	-1.51<br>(-1.68 to -1.34)
American Samoa	0.15（0.1-0.22）	7.19（4.75-10.47）	0.18（0.12-0.25）	3.53（2.37-4.99）	-2.31<br>(-2.83 to -1.78)
Andorra	0.54（0.33-0.86）	7.33（4.46-11.68）	0.72（0.41-1.15）	3.67（2.12-5.88）	-2.37<br>(-2.82 to -1.91)
Angola	42.05（24.98-64.37）	10.66（6.41-16.33）	100.42（66.31-149.47）	8.64（5.66-12.97）	-0.64<br>(-0.91 to -0.37)
Antigua and Barbuda	0.74（0.63-0.86）	10.43（8.84-12.17）	1.3（1.08-1.55）	9.97（8.3-11.91）	-0.69<br>(-2.39 to 1.04)

Argentina	703.68（577.21-849.47）	16.96（13.91-20.47）	716.34（575.74-873.21）	9.86（7.93-12.02）	-1.56<br>(-1.69 to -1.43)
Armenia	68.67（62.56-74.77）	20.57（18.64-22.54）	56.64（48.97-65.44）	9.6（8.29-11.09）	-2.59<br>(-3.96 to -1.21)
Australia	186.98（155.35-224.71）	7.24（6.01-8.69）	199.85（157.87-246.54）	3.23（2.56-3.98）	-2.78<br>(-2.89 to -2.67)
Austria	145.47（120.66-175.02）	9.23（7.65-11.1）	116.17（93.02-141.9）	4.87（3.92-5.94）	-1.8<br>(-2.04 to -1.57)
Azerbaijan	80.88（66.22-98.01）	14.01（11.41-17.1）	115.84（84.81-166.62）	9.57（6.96-13.72）	-1.17<br>(-1.41 to -0.93)
Bahamas	2.34（2.01-2.71）	13.28（11.4-15.37）	6.17（4.87-7.84）	13.1（10.36-16.58）	-0.01<br>(-0.59 to 0.58)
Bahrain	3.09（2.15-4.37）	20.38（14.16-28.91）	6.51（4.11-10.35）	9.36（6.06-14.34）	-2.44<br>(-3.12 to -1.76)
Bangladesh	1120.85（739.33-1638.18）	21.16（13.96-30.99）	2005.7（1268.13-3051.75）	12.29（7.77-18.71）	-1.58<br>(-1.86 to -1.3)
Barbados	3.38（2.95-3.89）	8.45（7.37-9.7）	6.38（4.91-8.11）	9.26（7.14-11.77）	0.46<br>(-0.71 to 1.65)
Belarus	246.05（200.84-297.02）	14.19（11.57-17.12）	225.37（164.95-296.99）	10.08（7.39-13.26）	-1.15<br>(-1.7 to -0.6)
Belgium	293.77（241.32-355.34）	14.34（11.78-17.33）	179.88（141.7-220.75）	5.75（4.57-7.05）	-2.82<br>(-3 to -2.64)
Belize	0.8（0.69-0.92）	7.06（6.05-8.11）	2.86（2.37-3.44）	8.67（7.16-10.4）	0.62<br>(-0.39 to 1.64)
Benin	13.26（8.79-19.07）	5.71（3.78-8.21）	33.45（21.48-50.16）	6.11（3.94-9.12）	0.21<br>(0.05 to 0.38)
Bermuda	1.09（0.87-1.35）	14.21（11.34-17.52）	1.54（1.16-2.03）	8.24（6.21-10.87）	-1.95<br>(-2.38 to -1.53)
Bhutan	3.43（2.07-5.5）	13.43（8.12-21.52）	7.82（4.91-11.83）	11.01（6.9-16.67）	-0.6<br>(-0.67 to -0.54)
Bolivia (Plurinational State of)	35.27（22.79-50.88）	9.98（6.46-14.4）	72.27（46.15-108.6）	6.94（4.44-10.38）	-1.17<br>(-1.24 to -1.09)
Bosnia and Herzegovina	96.71（78.69-117.06）	20（16.24-24.27）	125.99（89.03-162.72）	14.85（10.49-19.18）	-0.91<br>(-1.22 to -0.59)
Botswana	8.52（5.49-12.12）	13.06（8.48-18.61）	14.94（9.45-23.51）	9.23（5.92-14.3）	-1.07<br>(-1.33 to -0.81)
Brazil	1497.12（1380.08-1610.68）	14.6（13.39-15.74）	3603.25（3233.52-3945.57）	11.51（10.31-12.6）	-0.71<br>(-1.01 to -0.4)
Brunei Darussalam	1.45（1.01-2.02）	14.17（9.76-19.98）	1.76（1.24-2.44）	5.37（3.78-7.45）	-3.15<br>(-3.65 to -2.64)
Bulgaria	225.55（186.96-268.77）	13.77（11.36-16.48）	312.92（248.42-385.76）	16.23（12.9-20.02）	0.65<br>(-0.37 to 1.68)
Burkina Faso	27.95（18.17-40.88）	5.62（3.65-8.24）	70.23（44.34-105.01）	6.98（4.43-10.47）	0.73<br>(0.6 to 0.87)
Burundi	30.85（18.8-46.18）	11.43（6.97-17.18）	39.28（25.39-59.83）	7.63（4.96-11.59）	-1.29<br>(-1.38 to -1.19)
Cabo Verde	2.86（1.82-4.24）	9.57（6.07-14.19）	4.77（2.77-7.13）	9.08（5.3-13.52）	-0.16<br>(-1.18 to 0.87)
Cambodia	51.78（34.05-75.21）	10.58（6.99-15.31）	124.71（80.17-196.98）	9.11（5.87-14.36）	-0.46<br>(-0.54 to -0.39)
Cameroon	28.94（19.41-42.3）	5.98（4-8.72）	95.97（56.7-151.25）	7.28（4.37-11.43）	0.65<br>(0.55 to 0.75)
Canada	374.09（313-442.31）	8.79（7.35-10.39）	374.37（302.28-458.86）	3.76（3.04-4.6）	-2.7<br>(-3.43 to -1.96)
Central African Republic	14.3（8.25-21.88）	11.65（6.89-17.7）	19.61（11.22-30.01）	9.02（5.32-13.63）	-0.84<br>(-0.91 to -0.77)
Chad	15.87（10.35-23.63）	4.77（3.1-7.12）	49.73（30.13-75.61）	8.15（4.97-12.42）	1.75<br>(1.59 to 1.91)
Chile	101.59（83.6-122.83）	8.36（6.87-10.11）	116.91（93.26-142.79）	3.52（2.81-4.3）	-2.72<br>(-3.56 to -1.87)
China	8508.26（6873.36-10059.95）	9.23（7.49-10.88）	15209.79（11864.9-19183.03）	5.85（4.58-7.35）	-1.47<br>(-1.78 to -1.16)
Colombia	300.26（253.29-351.46）	15.55（13.09-18.24）	407.87（307.82-524.86）	5.94（4.49-7.64）	-3.06<br>(-3.65 to -2.47)
Comoros	2.13（1.36-3.1）	9.58（6.15-13.96）	3.87（2.55-5.7）	7.15（4.72-10.54）	-0.96<br>(-1.09 to -0.84)
Congo	13.44（8.12-19.8）	11.37（6.98-16.73）	23.86（16.05-34.86）	9.09（6.09-13.41）	-0.71<br>(-0.84 to -0.58)

Cook Islands	0.05（0.03-0.07）	3.68（2.46-5.18）	0.07（0.05-0.11）	2.19（1.45-3.35）	-1.67 (-2.05 to -1.28)
Costa Rica	22.29（18.15-26.95）	10.86（8.83-13.14）	31.19（24.15-39.68）	4.55（3.53-5.79）	-2.86 (-3.44 to -2.28)
Coted'Ivoire	29.11（19.07-42.4）	7.13（4.72-10.38）	71.36（44.15-116.5）	6.19（3.86-9.91）	-0.45 (-0.55 to -0.35)
Croatia	168.27（138.5-202.61）	21.81（17.95-26.33）	142.05（112.62-178.5）	11.76（9.31-14.8）	-1.87 (-2.41 to -1.34)
Cuba	328.54（273.9-392.34）	25.63（21.36-30.61）	692.67（542.65-882.85）	27.96（21.89-35.69）	0.16 (-0.62 to 0.95)
Cyprus	9.1（6.19-13.19）	10.13（6.79-14.98）	12.34（8.57-17.54）	4.53（3.14-6.44）	-2.69 (-3.13 to -2.25)
Czechia	219.12（183.19-260.85）	11.82（9.86-14.09）	192.94（154.48-239.86）	6.81（5.45-8.45）	-1.73 (-2.41 to -1.05)
Democratic People's Republic of Korea	92.49（58.53-134.31）	5.29（3.38-7.63）	178.6（117.02-256.58）	4.51（2.96-6.48）	-0.52 (-0.61 to -0.43)
Democratic Republic of the Congo	143.79（89.46-213.56）	8.89（5.58-13.27）	274.1（172.81-404.01）	7.34（4.65-10.88）	-0.63 (-0.75 to -0.51)
Denmark	113.74（94.73-134.43）	10.6（8.83-12.5）	87.97（71.1-106.4）	5.45（4.41-6.58）	-2.1 (-2.34 to -1.85)
Djibouti	1.37（0.85-2.15）	10.43（6.54-16.21）	5.9（3.61-8.96）	9.32（5.78-14.07）	-0.37 (-0.44 to -0.29)
Dominica	1.06（0.78-1.41）	13.58（9.93-18.06）	1.46（1-2.06）	13.85（9.49-19.45）	0.07 (-0.04 to 0.18)
Dominican Republic	39.75（28.82-54.81）	9.8（7.09-13.56）	107.64（69.97-159.07）	8.96（5.82-13.23）	-0.28 (-0.68 to 0.11)
Ecuador	39.32（32.57-47.02）	6.68（5.52-8）	69.64（51.03-93.2）	3.53（2.6-4.72）	-2.14 (-3.42 to -0.85)
Egypt	172.63（127.22-240.3）	6.59（4.81-9.16）	411.52（288.69-572.37）	6.61（4.66-9.2）	-0.02 (-0.29 to 0.26)
El Salvador	20.34（15.92-25.54）	5.85（4.57-7.34）	31.84（23.62-42.79）	4.03（2.99-5.43）	-1.06 (-1.46 to -0.66)
Equatorial Guinea	2.18（1.25-3.42）	10.35（6.06-16.1）	3.52（2-5.72）	7.13（4.09-11.56）	-1.19 (-1.39 to -0.99)
Eritrea	10.38（6.49-15.24）	9.58（6.07-14）	21.05（13.96-30.27）	7.67（5.11-11.02）	-0.72 (-0.81 to -0.63)
Estonia	31.07（25.55-37.15）	11.61（9.55-13.89）	23.07（17.64-29.04）	6.56（5.01-8.27）	-1.81 (-2.86 to -0.75)
Eswatini	4.43（2.6-6.6）	14.21（8.39-21.06）	7.77（4.44-11.57）	12.13（6.98-17.99）	-0.48 (-0.68 to -0.28)
Ethiopia	189.62（111.48-279.16）	8.52（5.1-12.49）	241.09（166.98-336.52）	5.18（3.59-7.22）	-1.61 (-1.65 to -1.56)
Fiji	1.51（1.03-2.22）	4.46（3.01-6.62）	3.47（2.27-5.12）	4.34（2.83-6.41）	-0.04 (-0.51 to 0.44)
Finland	40.4（33.29-48.62）	4.26（3.51-5.13）	41.39（32.84-51.34）	2.37（1.89-2.94）	-1.85 (-2.55 to -1.15)
France	1980.39（1642.63-2372.1）	18.36（15.24-21.98）	1324.18（1036.01-1646.86）	7.09（5.57-8.81）	-2.94 (-3.34 to -2.54)
Gabon	7.71（4.92-11.52）	11.26（7.17-16.92）	10.5（6.8-15.35）	9.16（5.97-13.37）	-0.63 (-0.82 to -0.43)
Gambia	1.04（0.7-1.49）	2.68（1.8-3.85）	2.73（1.79-3.95）	2.56（1.67-3.7）	-0.16 (-0.82 to 0.49)
Georgia	154.16（136.75-172.99）	18.78（16.62-21.09）	136.23（116.62-157.69）	16.96（14.52-19.62）	-0.07 (-0.91 to 0.78)
Germany	1200（1003.05-1427.89）	7.29（6.1-8.66）	1304.56（1042.27-1594.76）	5.12（4.12-6.23）	-1.1 (-1.33 to -0.87)
Ghana	30.71（20.28-45.62）	4.66（3.08-6.93）	116.1（70.43-172.81）	6.52（3.98-9.69）	1.1 (0.96 to 1.25)
Greece	337.71（297.69-377.83）	16.9（14.88-18.91）	322.8（274.57-369.33）	9.89（8.48-11.31）	-1.71 (-2.03 to -1.38)
Greenland	0.45（0.32-0.62）	12.88（9.11-17.68）	0.58（0.4-0.83）	6.92（4.76-10）	-1.83 (-2.18 to -1.48)
Grenada	0.83（0.67-1.03）	8.66（6.98-10.72）	0.91（0.71-1.14）	6.5（5.07-8.17）	-0.73 (-2.13 to 0.68)
Guam	0.26（0.19-0.33）	3.62（2.66-4.68）	0.35（0.27-0.46）	1.33（1.01-1.74）	-2.88 (-3.81 to -1.94)
Guatemala	36.82（32.79-41.06）	10.84（9.66-12.07）	39.29（32.11-47.44）	3.09（2.53-3.72）	-4.05 (-4.97 to -3.12)

Guinea	19.36（12.44-27.77）	4.92（3.15-7.08）	46.75（29.15-70.86）	7.48（4.69-11.29）	1.39 (1.28 to 1.49)
Guinea-Bissau	3.6（2.06-5.62）	8.05（4.68-12.44）	6.5（3.84-9.52）	8.77（5.27-12.84）	0.29 (0.22 to 0.36)
Guyana	2.93（2.33-3.71）	6.77（5.38-8.56）	4.35（3.06-5.94）	5.73（4.05-7.8）	-0.47 (-1.54 to 0.62)
Haiti	57.87（34.71-87.04）	16.09（9.72-24.23）	101.7（59.76-157.53）	13.7（8.08-21.33）	-0.49 (-0.6 to -0.38)
Honduras	16.13（11.51-22.08）	7.22（5.14-9.91）	66.28（42.84-94.37）	9.39（6.05-13.37）	0.9 (0.63 to 1.17)
Hungary	360.53（298-436.34）	18.21（15.06-22.05）	348.18（272.13-434.02）	13.59（10.59-16.96）	-0.81 (-1.29 to -0.33)
Iceland	1.73（1.42-2.09）	4.61（3.79-5.57）	1.63（1.28-2.01）	2.07（1.64-2.56）	-2.43 (-2.96 to -1.9)
India	7815.11（6208.23-9591.77）	15.41（12.14-18.99）	18415.32（15616.7-21554.61）	12.96（10.98-15.17）	-0.51 (-0.86 to -0.15)
Indonesia	638.94（453.56-808.38）	6.23（4.4-7.96）	1615.28（1073.96-2146.12）	6.33（4.2-8.44）	0.05 (-0.01 to 0.12)
Iran (Islamic Republic of)	431.82（353.58-503.72）	15.94（12.79-18.72）	939.08（798.41-1075.66）	10.98（9.28-12.6）	-1.2 (-1.3 to -1.09)
Iraq	121.19（82.39-171.04）	13.52（9.2-19.09）	302.93（197.3-435.09）	12.3（8.03-17.67）	-0.25 (-0.51 to 0.02)
Ireland	55.37（45.53-66.04）	10.2（8.37-12.18）	40.75（32.56-50.47）	3.88（3.1-4.8）	-2.93 (-3.38 to -2.49)
Israel	43.59（35.47-52.76）	6.96（5.64-8.42）	66.85（52.69-83）	4.05（3.2-5.03）	-1.79 (-2.35 to -1.23)
Italy	1983.96（1831.89-2129.53）	16.61（15.31-17.84）	1420.35（1242.19-1570.68）	7.03（6.23-7.74）	-2.71 (-2.9 to -2.52)
Jamaica	16.66（13.29-20.55）	7.11（5.67-8.77）	30.55（20.75-42.34）	7.94（5.39-11.01）	0.56 (0.18 to 0.95)
Japan	821.04（768.78-859.58）	3.9（3.63-4.09）	1046.36（910.1-1128.91）	1.8（1.6-1.92）	-2.44 (-2.92 to -1.95)
Jordan	11.41（7.64-16.34）	8.7（5.85-12.49）	29.65（19.28-44.49）	4.04（2.64-6.04）	-2.39 (-2.77 to -2.02)
Kazakhstan	229.22（204.77-257.28）	14.5（12.92-16.3）	137.62（117.64-158.24）	6.03（5.15-6.93）	-2.69 (-3.52 to -1.86)
Kenya	57.5（41.01-78.04）	6.31（4.51-8.57）	175.5（128.43-223.75）	6.97（5.11-8.88）	0.33 (0.21 to 0.44)
Kiribati	0.05（0.03-0.07）	1.23（0.81-1.78）	0.09（0.06-0.14）	1.28（0.81-1.94）	0.11 (0.03 to 0.19)
Kuwait	4.62（3.68-5.74）	8.36（6.62-10.4）	6.4（4.68-8.4）	2.55（1.86-3.34）	-3.66 (-7.07 to -0.12)
Kyrgyzstan	34.23（27-42.79）	9.05（7.15-11.31）	23.02（16.88-29.68）	4.03（2.96-5.19）	-2.7 (-3.79 to -1.59)
Lao People's Democratic Republic	23.93（15.44-35.83）	10.13（6.56-15.11）	34.26（21.39-53.27）	7.04（4.42-10.93）	-1.16 (-1.23 to -1.1)
Latvia	63.45（52.77-76.18）	13.57（11.28-16.3）	44.55（34.87-56.05）	8.57（6.71-10.78）	-1.41 (-2.41 to -0.39)
Lebanon	42.21（26.66-62.28）	17.01（10.85-24.99）	86.99（60-123.42）	11.15（7.7-15.82）	-1.31 (-1.56 to -1.07)
Lesotho	11.08（7.32-16.01）	10.91（7.23-15.76）	19.87（12.54-29.04）	14.9（9.44-21.72）	1.06 (0.79 to 1.34)
Liberia	7.4（4.65-11.81）	5.52（3.47-8.8）	12.77（7.8-20.11）	6.25（3.84-9.81）	0.42 (0.16 to 0.68)
Libya	37.86（24.86-57.78）	18.17（11.94-27.68）	90.28（58.78-135.29）	17.12（11.18-25.62）	-0.16 (-0.49 to 0.17)
Lithuania	85.22（70.29-102.64）	14.69（12.11-17.71）	82.1（63.76-103.64）	11.24（8.72-14.2）	-0.68 (-1.75 to 0.4)
Luxembourg	9.69（8.51-11.05）	13.58（11.92-15.45）	7.59（6.39-8.76）	5.55（4.68-6.41）	-2.94 (-3.28 to -2.61)
Madagascar	47.75（31.34-67.59）	8.37（5.51-11.86）	62.72（41.02-92.49）	5.63（3.7-8.26）	-1.28 (-1.34 to -1.22)
Malawi	13.35（9.14-18.94）	3.09（2.12-4.39）	23.99（16.32-35.75）	2.95（2.01-4.37）	-0.14 (-0.29 to 0.01)
Malaysia	99（65.01-144.55）	9.73（6.37-14.25）	254.56（172.69-358.15）	7.7（5.21-10.84）	-0.92 (-1.24 to -0.6)
Maldives	0.66（0.42-1）	8.05（5.11-12.32）	1.05（0.7-1.52）	3.22（2.15-4.67）	-2.99 (-3.22 to -2.76)

Mali	25.98（18.39-36.06）	5.92（4.18-8.23）	51.94（33.88-77.2）	5.52（3.6-8.19）	-0.18<br>(-0.35 to -0.01)
Malta	6.35（5.09-7.76）	11.62（9.3-14.19）	5.66（4.44-7.01）	4.18（3.28-5.18）	-3.29<br>(-3.59 to -2.98)
Marshall Islands	0.08（0.05-0.13）	4.85（2.96-7.5）	0.14（0.08-0.22）	4.86（2.83-7.58）	0.06<br>(-0.19 to 0.3)
Mauritania	5.99（4.02-8.63）	5.27（3.53-7.62）	14.56（8.94-22.39）	6.1（3.75-9.36）	0.49<br>(0.2 to 0.78)
Mauritius	10.19（8.81-11.77）	11.8（10.2-13.63）	20.01（17.23-22.97）	8.54（7.36-9.81）	-1.18<br>(-3.08 to 0.75)
Mexico	612.56（584.88-638.31）	13.59（12.93-14.17）	761.72（650.45-883.79）	5.13（4.39-5.94）	-3.11<br>(-3.49 to -2.74)
Micronesia (Federated States of)	0.27（0.17-0.42）	4.89（3.13-7.58）	0.29（0.19-0.45）	3.99（2.53-6.07）	-0.66<br>(-0.74 to -0.58)
Monaco	2.79（1.82-4.02）	29.08（18.96-42.04）	2.37（1.61-3.38）	17.74（12.11-25.38）	-1.59<br>(-1.69 to -1.49)
Mongolia	9.37（6.5-12.8）	7.59（5.26-10.4）	9.91（6.84-14.12）	4.05（2.8-5.77）	-2.08<br>(-2.76 to -1.4)
Montenegro	18.84（14.62-24.11）	24.39（18.93-31.23）	33.02（25.32-43.2）	25.17（19.28-32.86）	0.19<br>(-0.08 to 0.47)
Morocco	186.15（125.28-265.13）	11.21（7.53-16）	428.57（271.45-639.37）	10.57（6.68-15.78）	-0.2<br>(-0.29 to -0.11)
Mozambique	65.03（41.87-94.61）	9.98（6.46-14.52）	110.62（73.12-159.04）	9.29（6.15-13.34）	-0.2<br>(-0.32 to -0.09)
Myanmar	248.75（152.74-369.92）	9.52（5.92-14.09）	307.27（195.03-452.65）	5.57（3.56-8.2）	-1.72<br>(-1.78 to -1.67)
Namibia	10.1（7.08-13.98）	13.09（9.17-18.15）	19.32（12.88-27.77）	12.48（8.38-17.9）	-0.14<br>(-0.27 to 0)
Nauru	0.03（0.02-0.04）	6.77（4.13-10.17）	0.02（0.01-0.04）	4.54（2.72-6.86）	-1.28<br>(-1.39 to -1.18)
Nepal	164.63（103.15-249.24）	16.3（10.19-24.73）	343.92（221.08-521.58）	12.61（8.09-19.12）	-0.8<br>(-0.9 to -0.7)
Netherlands	202.05（168.48-241.12）	7.7（6.43-9.19）	192.62（156.06-234.11）	3.99（3.24-4.84）	-2.14<br>(-2.37 to -1.91)
New Zealand	29.28（24.18-35）	5.66（4.66-6.77）	27.56（22.2-33.48）	2.4（1.93-2.91）	-2.74<br>(-3.38 to -2.1)
Nicaragua	12.83（9.2-17.41）	7.75（5.55-10.52）	26.56（18.29-38.05）	4.76（3.27-6.81）	-1.7<br>(-1.92 to -1.49)
Niger	15.46（9.7-23.03）	5.16（3.26-7.67）	51.34（30.39-80.77）	6.02（3.6-9.41）	0.51<br>(0.32 to 0.69)
Nigeria	380.32（266.13-514.94）	7.47（5.3-10.04）	604.38（427.57-820.43）	6.62（4.74-8.9）	-0.39<br>(-0.45 to -0.34)
Niue	0.01（0.01-0.01）	3.31（2.14-4.91）	0.01（0.01-0.01）	3.15（2.05-4.64）	-0.17<br>(-0.26 to -0.07)
North Macedonia	47.79（37.21-61.45）	21.14（16.43-27.34）	76.78（56.58-102.78）	17.61（13.05-23.4）	-0.62<br>(-0.88 to -0.37)
Northern Mariana Islands	0.08（0.05-0.13）	7.26（4.62-10.91）	0.22（0.14-0.31）	4.34（2.88-6.24）	-1.7<br>(-2.16 to -1.24)
Norway	39.92（36.23-43.35）	4.24（3.85-4.6）	34.37（29.88-38.2）	2.48（2.17-2.76）	-1.46<br>(-2.65 to -0.26)
Oman	3.28（2.08-4.93）	4.65（2.96-6.98）	4.74（3.05-7.06）	2.66（1.68-3.96）	-1.74<br>(-2.34 to -1.14)
Pakistan	1839.1（1378.24-2419.12）	28.43（21.18-37.55）	3255.56（2301.2-4457.4）	25.52（18.05-34.84）	-0.34<br>(-0.43 to -0.26)
Palau	0.04（0.02-0.06）	3.62（2.3-5.39）	0.06（0.04-0.1）	2.75（1.71-4.32）	-0.98<br>(-1.17 to -0.79)
Palestine	8.73（5.75-12.82）	9.05（5.97-13.27）	14.5（9.99-20.62）	5.74（3.96-8.16）	-1.48<br>(-1.68 to -1.28)
Panama	18.72（16.19-21.33）	10.83（9.36-12.34）	26.52（19.76-33.23）	4.8（3.58-6.02）	-2.57<br>(-2.98 to -2.15)
Papua New Guinea	5.63（3.32-8.93）	3.49（2.07-5.61）	14.21（8.75-22.76）	3.26（1.98-5.26）	-0.18<br>(-0.3 to -0.05)
Paraguay	18.89（13.27-26.05）	7.24（5.08-9.98）	64.28（41.15-95.88）	9.21（5.91-13.72）	0.82<br>(0.35 to 1.29)
Peru	100.87（72.64-135.58）	7.52（5.41-10.12）	150.69（98.23-221.1）	3.72（2.42-5.46）	-2.16<br>(-3.03 to -1.28)
Philippines	163.84（134.98-204.47）	5.43（4.48-6.76）	458.64（369.89-553.08）	4.97（4.02-5.98）	-0.26<br>(-0.48 to -0.04)

Poland	1074.59（1018.9-1130.37）	18.6（17.61-19.58）	1416.85（1260.67-1568.51）	14.35（12.77-15.89）	-0.87 (-1.23 to -0.5)
Portugal	322.46（266.78-391.68）	17.43（14.42-21.17）	276.46（217.55-344.57）	8.54（6.74-10.63）	-2.42 (-2.66 to -2.18)
Puerto Rico	64.39（51.95-78.04）	13.91（11.22-16.86）	48.1（36.1-62.76）	4.86（3.66-6.36）	-3.41 (-4.82 to -1.99)
Qatar	1.33（0.87-1.99）	18.86（12.48-28.32）	4.84（3.03-7.58）	8.81（5.58-13.74）	-2.43 (-3.63 to -1.21)
Republic of Korea	441.5（293.11-586.71）	14.21（9.4-18.91）	448.73（311.06-611.34）	3.72（2.58-5.08）	-4.23 (-4.47 to -4)
Republic of Moldova	87.93（77.22-99.75）	15.51（13.61-17.59）	97.59（82.54-114.34）	11.85（10.03-13.88）	-0.85 (-2.16 to 0.48)
Romania	476.86（390.58-575.94）	13.05（10.68-15.77）	731.92（576.48-915.07）	14.73（11.59-18.44）	0.42 (-0.2 to 1.03)
Russian Federation	3294.57（3160.25-3418.24）	13.47（12.9-14）	2833.95（2508.51-3130.96）	8.51（7.54-9.4）	-1.39 (-1.79 to -0.99)
Rwanda	43.97（28.25-65.1）	13.57（8.72-20.14）	55.85（35.14-86.49）	8.02（5.09-12.39）	-1.69 (-1.76 to -1.62)
Saint Kitts and Nevis	0.48（0.41-0.55）	8.81（7.54-10.2）	0.78（0.62-0.98）	9.38（7.42-11.68）	0.31 (-0.96 to 1.6)
Saint Lucia	1.55（1.35-1.77）	14.22（12.39-16.24）	3.59（2.79-4.46）	12.09（9.42-15.02）	-0.52 (-1.18 to 0.15)
Saint Vincent and the Grenadines	1.28（1.1-1.48）	13.83（11.84-15.96）	2.73（2.29-3.24）	15.16（12.75-18）	0.2 (-0.71 to 1.11)
Samoa	0.16（0.11-0.22）	1.61（1.12-2.3）	0.23（0.15-0.33）	1.38（0.94-2.04）	-0.47 (-0.56 to -0.39)
San Marino	0.59（0.39-0.83）	12.5（8.38-17.65）	0.48（0.27-0.76）	4.79（2.69-7.69）	-3.42 (-3.83 to -3)
Sao Tome and Principe	0.34（0.22-0.5）	4.34（2.81-6.36）	0.59（0.38-0.87）	5.16（3.32-7.67）	0.53 (0.2 to 0.87)
Saudi Arabia	31.02（20.28-44.82）	5.23（3.42-7.56）	54.4（36.88-78.3）	3.5（2.36-5.06）	-1.31 (-1.45 to -1.17)
Senegal	21.56（14.27-30.95）	5.84（3.86-8.4）	59.84（36.1-90.69）	6.99（4.23-10.61）	0.6 (0.4 to 0.81)
Serbia	293.11（204.21-423.04）	21.04（14.66-30.39）	343.83（235.22-476.38）	15.19（10.39-21.05）	-0.99 (-1.18 to -0.81)
Seychelles	2.08（1.44-2.88）	28.76（19.82-39.69）	3.04（2.07-4.3）	22.79（15.57-32.21）	-0.83 (-1.36 to -0.3)
Sierra Leone	13.81（9.05-20.44）	5.75（3.75-8.53）	26.11（16.77-39.79）	6.44（4.15-9.79）	0.38 (0.27 to 0.49)
Singapore	24.92（20.33-30.24）	10.07（8.2-12.23）	30.9（24.3-38.68）	2.94（2.31-3.68）	-3.89 (-4.93 to -2.84)
Slovakia	130.58（93.34-175.65）	16.38（11.71-22.06）	133.7（92.39-190.47）	10.32（7.13-14.7）	-1.5 (-1.55 to -1.45)
Slovenia	39.39（32.42-47.2）	12.43（10.22-14.92）	40.46（31.85-50.57）	6.9（5.45-8.61）	-2.08 (-3 to -1.15)
Solomon Islands	0.65（0.38-1.01）	5.18（3.14-8.06）	1.33（0.83-2.01）	4.17（2.62-6.33）	-0.7 (-0.83 to -0.57)
Somalia	23.88（14.22-37.02）	10.84（6.54-16.75）	52.88（32.34-80.1）	8.22（5.09-12.39）	-0.87 (-0.95 to -0.79)
South Africa	205.16（158.98-295.93）	8.62（6.7-12.39）	437.22（379.1-498.1）	7.94（6.87-9.06）	-0.3 (-0.66 to 0.06)
South Sudan	33.16（20.19-50.51）	10.69（6.52-16.29）	32.03（19.01-50.03）	8.37（4.99-13.01）	-0.79 (-0.92 to -0.66)
Spain	1520.06（1247.88-1822）	20.77（17.05-24.9）	1156.82（899.8-1448.57）	9.08（7.09-11.34）	-2.7 (-3.14 to -2.25)
Sri Lanka	63（44.45-88.87）	5.53（3.88-7.83）	189.17（107.68-300.14）	5.53（3.19-8.75）	-0.04 (-0.59 to 0.52)
Sudan	141.4（80.92-244.29）	13.62（7.72-23.94）	216.21（131.15-333.91）	10.94（6.63-16.92）	-0.7 (-0.76 to -0.65)
Suriname	1.48（1.08-2）	5.11（3.72-6.86）	3.33（2.08-4.98）	4.34（2.72-6.5）	-0.43 (-1.25 to 0.39)
Sweden	65.11（54.64-77.03）	3.11（2.61-3.67）	57.81（45.96-71.05）	1.88（1.5-2.31）	-1.51 (-2.81 to -0.18)
Switzerland	102.87（85.31-123.13）	7.61（6.32-9.1）	80.25（62.74-99.4）	3.28（2.58-4.06）	-2.8 (-3.23 to -2.36)
Syrian Arab Republic	47.67（34.2-67.22）	8.66（6.21-12.25）	96.29（67.22-133.31）	7.13（5.04-9.76）	-0.7 (-1.07 to -0.33)

Taiwan (Province of China)	130.39（108.13-155.91）	6.82（5.64-8.18）	187.41（147.4-235.28）	3.35（2.64-4.2）	-2.16 (-2.64 to -1.68)
Tajikistan	25.09（18.47-33.01）	7.76（5.73-10.22）	28.78（18.74-40.61）	4.62（2.92-6.56）	-1.72 (-2.1 to -1.34)
Thailand	438.24（313.68-608.8）	11.75（8.38-16.33）	943.72（622.29-1396.51）	6.79（4.48-10.04）	-1.77 (-2.07 to -1.46)
Timor-Leste	1.54（0.97-2.36）	6.27（3.99-9.53）	5.81（3.66-8.74）	5.71（3.6-8.62）	-0.28 (-0.41 to -0.15)
Togo	7.7（5.05-11.4）	5.92（3.89-8.76）	28.06（16.89-42.01）	7.06（4.32-10.55）	0.56 (0.36 to 0.76)
Tokelau	0.01（0-0.01）	4.17（2.56-6.58）	0.01（0-0.01）	2.99（1.88-4.67）	-1.07 (-1.17 to -0.97)
Tonga	0.22（0.14-0.35）	3.68（2.31-5.81）	0.31（0.19-0.48）	3.26（2.02-5.05）	-0.38 (-0.69 to -0.06)
Trinidad and Tobago	8.6（7.51-9.85）	8.43（7.37-9.64）	16.25（11.99-21）	6.41（4.74-8.27）	-0.76 (-1.08 to -0.45)
Tunisia	93.65（62.29-135.62）	16.32（10.86-23.62）	194.99（118.33-301.94）	12.06（7.34-18.61）	-1.02 (-1.11 to -0.93)
Turkey	808.81（526.2-1237.5）	21.1（13.75-32.25）	1257.35（844.76-1798.59）	11.07（7.45-15.81）	-2.07 (-2.52 to -1.62)
Turkmenistan	27.19（23.98-30.31）	11.99（10.53-13.42）	24.27（18.76-31.36）	5.2（4.03-6.7）	-2.61 (-3.13 to -2.08)
Tuvalu	0.03（0.02-0.04）	4.14（2.73-6）	0.04（0.03-0.06）	3.55（2.33-5.23）	-0.49 (-0.55 to -0.43)
Uganda	87.22（58.79-122.37）	12.04（8.14-16.86）	144.18（92.81-216.25）	9.39（6.08-13.98）	-0.81 (-0.92 to -0.71)
Ukraine	1252.11（1028.29-1499.12）	12.7（10.43-15.22）	841.02（544.99-1234.99）	7.88（5.11-11.56）	-1.51 (-2.16 to -0.86)
United Arab Emirates	3.39（1.96-5.37）	10.4（6.13-16.35）	13.93（9.17-20.16）	7.35（4.89-10.56）	-1.21 (-3.19 to 0.82)
United Kingdom	863.2（826.25-891.96）	7.05（6.75-7.29）	800.06（734.17-844.83）	4.5（4.16-4.74）	-1.47 (-1.99 to -0.95)
United Republic of Tanzania	124.51（81.22-182.5）	9.95（6.52-14.57）	183.49（115.93-291.48）	6.67（4.24-10.5）	-1.29 (-1.39 to -1.19)
United States of America	0.91（0.63-1.31）	7.65（7.23-7.95）	1.47（0.93-2.2）	4.81（4.42-5.07）	-1.7 (-2.17 to -1.24)
United States Virgin Islands	3249.29（3069.97-3376.79）	9.62（6.66-13.78）	3813.44（3501.2-4026.91）	5.82（3.66-8.68）	-1.56 (-1.75 to -1.38)
Uruguay	133.54（108.64-162.72）	25.78（20.97-31.42）	104.45（83.52-129.03）	14.42（11.55-17.8）	-1.9 (-2.02 to -1.77)
Uzbekistan	136.06（112.36-161.92）	9.88（8.17-11.74）	104.23（79.58-133.51）	3.58（2.73-4.56）	-3.32 (-4.07 to -2.57)
Vanuatu	0.22（0.13-0.35）	3.8（2.28-5.93）	0.51（0.32-0.78）	3.06（1.92-4.7）	-0.69 (-0.83 to -0.55)
Venezuela (Bolivarian Republic of)	179.89（158.57-200.86）	16.74（14.7-18.7）	437.89（323.79-580.87）	11.94（8.86-15.8）	-1.21 (-1.53 to -0.89)
Viet Nam	342.63（231.74-498.12）	7.04（4.77-10.22）	859.22（549.84-1272.12）	7.38（4.78-10.87）	0.17 (0.02 to 0.32)
Yemen	77.12（45.79-122.87）	14.43（8.49-23.08）	203.74（117.24-313.95）	14.07（8.07-21.81）	-0.06 (-0.18 to 0.06)
Zambia	34.86（23.09-50.31）	11.46（7.59-16.57）	73.38（37.02-158.39）	10.32（5.33-21.67）	-0.31 (-0.52 to -0.09)
Zimbabwe	52.97（36-74.62）	11.32（7.72-15.9）	81.48（56.17-115.28）	10.78（7.44-15.21）	-0.13 (-0.31 to 0.06)

Abbreviations: SDI: Sociodemographic Index; ASMR: age-standardised mortality rate; AAPC=average annual percentage change; CI = confidence interval; UI = uncertainty interval.

Table S3. The DALYs and ASDR of older adults with larynx cancer in 1990 and 2021, and its AAPC from 1990 to 2021

Characteristics	1990		2021		1990-2021
	DALYs (95 % UI)	ASDR (95 % UI)	DALYs (95 % UI)	ASDR (95 % UI)	AAPC (95 % CI)
Global	214695.24（179808.97-254988.32）	295.88（247.28-351.69）	429895.63（379221.77-494084.68）	245.19（216.08-281.95）	-0.59 (-0.9 to -0.27)
High SDI	275857.16（247845.2-300780.59）	224.91（201.85-245.11）	536503.97（478646.19-602011.58）	160.4（142.92-179.92）	-1.08 (-1.2 to -0.96)
High-middle SDI					
Low SDI	3543.93（2893.5-4333.66）	150.2（122.47-183.8）	5691.06（4216.3-7464.68）	79.32（58.8-104.01）	-2.03 (-2.58 to -1.48)
Low-middle SDI	4747.8（4050.1-5572.74）	152.41（129.99-178.8）	4299.34（3515.8-5174.24）	60.88（49.86-73.24）	-3.11 (-3.81 to -2.4)
Middle SDI	11362.84（10020.32-12980.42）	352.35（310.49-402.57）	22030.89（18110.55-26702.15）	328.77（270.28-398.51）	-0.18 (-0.88 to 0.52)
Region	18328.68（17205.86-19503.38）	305.81（286.68-325.78）	15015.5（13369.01-16845.27）	145.01（129.12-162.57）	-2.4 (-2.75 to -2.05)
Andean Latin America	75339.24（70696.5-80461.62）	370.2（347.11-395.48）	89837.79（81534.57-98668.31）	303.74（275.65-333.66）	-0.62 (-0.8 to -0.45)
Australasia	24748.34（23330.22-26115.75）	260（244.55-274.48）	36556.5（31737.95-41940.85）	118.86（103.23-136.28）	-2.58 (-3.05 to -2.11)
Caribbean	5249.46（3497.61-7375.92）	200.12（134.53-280.8）	10142.2（7160.36-13769.54）	165.92（117.08-226.71）	-0.6 (-0.76 to -0.45)
Central Asia	192878.09（156341.64-228310.26）	183.41（149-216.49）	320417.43（249150.1-405325.33）	114.4（89.14-144.3）	-1.53 (-1.8 to -1.26)
Central Europe	124185.24（117311.54-131167.86）	317.6（299.87-335.7）	98425.57（86958.13-110653.88）	199.38（176.23-224.09）	-1.41 (-2 to -0.82)
Central Latin America	17685.57（13865.11-21739.41）	199.04（156.23-244.4）	27892.46（22134.19-35246.07）	145.68（115.71-183.19）	-1 (-1.05 to -0.95)

Central Sub-Saharan Africa	27257.03（23600.96-30771.12）	107.37（92.99-121.1）	27073.38（23117.94-30795.84）	43.16（37.03-49.26）	-2.9<br>(-3.27 to -2.54)
East Asia	79908.35（76008.72-83327.17）	173.94（165.53-181.32）	89687.59（83515.5-94806.12）	101.76（94.84-107.51）	-1.77<br>(-1.92 to -1.62)
Eastern Europe	55706.33（45348.49-68882.68）	282.87（229.51-351.12）	101386.19（85626.71-118656.33）	195.55（164.92-228.7）	-1.19<br>(-1.28 to -1.09)
Eastern Sub-Saharan Africa	224.25（159.88-313.42）	71.78（51.46-100.29）	483.37（347.51-678.7）	62.95（45.18-88.74）	-0.39<br>(-0.46 to -0.31)
High-income Asia Pacific	252985.21（208564.78-303380.32）	373.2（306.46-448.33）	530514.48（457311.82-614719.62）	290.09（249.94-336.24）	-0.73<br>(-1.04 to -0.42)
High-income North America	46295.48（38931.32-54387.69）	156.46（131.31-184.03）	105774.71（88396.38-127103.52）	132.19（110.42-159.09）	-0.54<br>(-0.62 to -0.47)
North Africa and Middle East	21045.29（18053.04-24374.95）	349.65（299.84-405.01）	19664.75（16595.97-23120.95）	175.73（148.35-206.59）	-2.05<br>(-2.23 to -1.86)
Oceania	6562.67（5259.69-8780.14）	200.38（160.78-267.61）	13403.13（11683.87-15192.98）	186.49（162.46-211.53）	-0.24<br>(-0.51 to 0.04)
South Asia	33931.07（31442.22-36444.03）	305.75（282.51-328.73）	79784.49（72225.89-87150.53）	244.58（221.23-267.21）	-0.68<br>(-0.93 to -0.43)
Southeast Asia	200858.31（187708.51-213663.2）	267.2（249.76-284.17）	148996.91（133519.07-163031.71）	128.32（115.65-140.26）	-2.37<br>(-2.53 to -2.21)
Southern Latin America	14904.69（11734.29-18674.56）	140.89（111.43-175.75）	29995.81（23990.81-36401.92）	136.23（109.39-164.65）	-0.1<br>(-0.14 to -0.06)
Southern Sub-Saharan Africa					
Tropical Latin America	3293.59（1713.29-5281.73）	382.18（201.19-609.66）	2566.82（1395.18-3967.66）	312（167.86-486.89）	-0.63<br>(-0.69 to -0.57)
Western Europe	977.5（717.54-1315.12）	393.09（288.53-529.23）	1393.28（945.66-1996.72）	231.12（156.91-331.15）	-1.74<br>(-2.22 to -1.26)
Western Sub-Saharan Africa	3060.88（2075.79-4504.19）	218.93（148.18-322.57）	5605.64（3756.76-8119.53）	137.87（92.18-200.02）	-1.5<br>(-1.62 to -1.38)
204 countries and territories	3.15（2.09-4.62）	134.62（89.23-196.83）	3.5（2.36-4.96）	64.65（43.56-91.47）	-2.38<br>(-2.91 to -1.85)
Afghanistan	11.89（7.2-19.04）	157.56（95.58-252.05）	15.18（8.68-24.31）	79.19（45.25-126.76）	-2.35<br>(-2.77 to -1.94)
Albania	1002.95（590.53-1535.38）	230.05（136.9-351.79）	2347.77（1553.06-3486.16）	181.98（119.88-271.33）	-0.71<br>(-0.93 to -0.49)
Algeria	14.98（12.69-17.49）	218.86（185.59-255.54）	27.74（23.09-33.19）	205.24（170.68-245.52）	-0.77<br>(-2.58 to 1.07)
American Samoa	15834.31（12959.44-19077.48）	372.66（305.02-449.01）	15153.68（12263.06-18461.78）	211.49（171.21-257.71）	-1.76<br>(-2.12 to -1.39)
Andorra	1661.74（1525.29-1803.42）	467.52（427.23-510.32）	1286.3（1109.2-1491.29）	211.15（182.16-244.75）	-2.7<br>(-4.08 to -1.3)
Angola	4114.31（3413.76-4933.31）	158.43（131.44-189.87）	3756.84（2998.62-4601.96）	63.29（50.59-77.52）	-3.14<br>(-3.25 to -3.03)
Antigua and Barbuda	3089.72（2580.8-3710.18）	201.34（168.15-241.8）	2309.82（1863.36-2812.21）	103.08（83.36-125.37）	-1.93<br>(-2.19 to -1.68)
Argentina	1935.85（1592.45-2330.14）	319.79（261.78-387.07）	2797.74（2057.94-4030.15）	211.79（155.09-304.41）	-1.29<br>(-1.49 to -1.09)
Armenia	52.26（44.82-60.41）	292.36（250.74-337.89）	134.87（105.92-172.17）	270.92（213.3-345.1）	0.01<br>(-0.12 to 0.15)
Australia	68.9（48.05-97.2）	399.65（278.52-565.2）	148.2（92.75-236.71）	174.73（112.24-270.51）	-2.62<br>(-3.16 to -2.08)
Austria	25395.81（16628.05-37026.59）	462.18（303.23-674.35）	43069.06（27228.62-65763.78）	252.45（159.66-385.31）	-1.76<br>(-2.04 to -1.48)
Azerbaijan	66.07（57.72-76.07）	172.61（150.95-198.54）	127.98（97.62-163.56）	183.62（140.18-234.54）	0.22<br>(-0.75 to 1.2)
Bahamas	5971.84（4872.25-7219.04）	334.29（272.53-404.02）	5522.89（4048.67-7347.77）	242.93（178.33-322.74）	-1.07<br>(-1.65 to -0.48)
Bahrain	6248.43（5127.89-7551.19）	308.97（253.66-373.12）	3636.82（2894.29-4459.07）	124.06（99.22-151.99）	-2.91<br>(-3.11 to -2.71)
Bangladesh	16.87（14.51-19.35）	147.82（127.09-169.52）	61.49（51.04-73.89）	177.55（147.2-213.27）	0.57<br>(-0.45 to 1.61)
Barbados	289.85（191.85-416.98）	120.88（79.98-173.92）	745.93（475.71-1122.53）	127.94（81.84-191.88）	0.2<br>(0.02 to 0.38)
Belarus	23.02（18.32-28.48）	291.88（232.29-361.07）	29.97（22.58-39.63）	165.28（124.46-218.65）	-2.07<br>(-2.53 to -1.61)
Belgium	80.01（48.04-128.74）	288.05（173.66-462.53）	161.17（101.71-243.59）	221.57（139.7-335）	-0.81<br>(-0.87 to -0.75)

Belize	750.65（484.08-1079.69）	200.12（129.21-287.98）	1484.47（941.44-2250.01）	134.31（85.36-202.89）	-1.27 (-1.34 to -1.2)
Benin	2284.45（1861.13-2764.67）	444.75（361.62-538.83）	2764.29（1949.62-3584.52）	324.45（228.81-420.62）	-0.99 (-1.34 to -0.64)
Bermuda	200.86（129.16-286.71）	285.2（184.25-406.83）	346.13（215.97-553.03）	199.32（125.81-314.01）	-1.11 (-1.3 to -0.92)
Bhutan	33522.63（31020.23-36023.81）	309.59（285.68-333.04）	78385.75（70833.54-85629.85）	245.72（221.87-268.47）	-0.71 (-0.96 to -0.45)
Bolivia (Plurinational State of)	29.9（20.95-41.76）	280.17（195.36-392.68）	37.83（26.56-52.38）	101.22（71.04-140.34）	-3.3 (-3.73 to -2.87)
Bosnia and Herzegovina	5270.89（4385.51-6259.03）	301.63（250.21-359.1）	6987.84（5572.22-8594.72）	373.04（297.49-458.65）	0.78 (-0.22 to 1.79)
Botswana	641.92（415.44-938.7）	120.12（77.86-175.8）	1565.86（979.38-2339.21）	146.02（91.73-218.4）	0.67 (0.49 to 0.84)
Brazil	699.83（425.14-1044.69）	250.01（151.95-374.02）	913.59（586.29-1395.8）	162.71（105.09-248.06）	-1.37 (-1.45 to -1.29)
Brunei Darussalam	55.59（35.53-82.33）	188.65（120.19-279.84）	101.01（57.97-152.27）	184.37（106.37-276.44）	-0.09 (-1.26 to 1.08)
Bulgaria	1177.05（770.5-1716.84）	223.47（146.94-324.82）	2751.16（1762.71-4352.71）	185.47（119.06-293.2）	-0.59 (-0.68 to -0.5)
Burkina Faso	667.15（448.15-978.92）	126.45（84.9-185.06）	2216.61（1289.11-3514.88）	154.5（90.96-243.82）	0.65 (0.56 to 0.74)
Burundi	8198.27（6913.94-9675.68）	192.56（162.41-227.16）	7405.28（6026.87-9005.29）	76.38（62.22-92.78）	-2.83 (-3.54 to -2.11)
Cabo Verde	348.76（199.45-536.32）	257.56（150-393.27）	480.7（271.21-741）	197.68（114.27-301.29）	-0.86 (-0.94 to -0.79)
Cambodia	346.77（226.73-516.9）	101.32（66.06-151.18）	1133.7（681.63-1724.25）	172.82（104.48-262.9）	1.78 (1.63 to 1.92)
Cameroon	2225.42（1837.21-2685.44）	178.55（147.32-215.54）	2355.99（1898.52-2872.19）	71.05（57.25-86.63）	-2.87 (-3.63 to -2.1)
Canada	187752.58（151215.33-223286.99）	185.53（149.84-219.98）	312529.84（241452.04-397935.38）	115.6（89.48-146.75）	-1.53 (-1.82 to -1.24)
Central African Republic	6281.89（5320.42-7335.56）	310.11（262.18-362.58）	7848.81（5916.71-10121.66）	113.98（85.99-146.94）	-3.18 (-3.77 to -2.58)
Chad	49.38（31.54-72.11）	207.06（132.57-301.96）	84.65（55.64-125.36）	148.64（97.78-219.94）	-1.09 (-1.24 to -0.94)
Chile	312.34（186.13-461.98）	241.41（145.69-356）	548.09（367.11-800.78）	189.26（126.94-277.81）	-0.79 (-0.94 to -0.64)
China	1（0.67-1.38）	69.65（46.8-97.05）	1.4（0.93-2.13）	41.33（27.55-62.76）	-1.67 (-2.05 to -1.29)
Colombia	446.34（364.91-537.75）	214.07（174.9-258.03）	598.68（464.11-758.69）	86.57（67.15-109.77）	-2.99 (-3.62 to -2.36)
Comoros	701.53（456.62-1025.48）	155.91（102.37-227.49）	1673.59（1029.53-2775.52）	133.67（82.76-218.31）	-0.49 (-0.6 to -0.38)
Congo	3837.65（3171.82-4616.12）	470.51（388.71-567.19）	3031.19（2391.8-3816.36）	258.56（203.48-326）	-1.85 (-2.39 to -1.3)
Cook Islands	6611.38（5540.74-7882.03）	513.17（430.04-611.62）	14126.88（11073.3-18035.46）	586.74（459.63-749.81）	0.37 (-0.39 to 1.13)
Costa Rica	184.36（125.24-266.04）	184.95（124.57-269.67）	254.37（176.9-362.51）	92.92（64.56-132.53）	-2.3 (-2.6 to -1.99)
Coted'Ivoire	4961.17（4153.35-5886.19）	265.92（222.31-315.6）	4206.33（3380.99-5215.31）	154.5（124.27-191.37）	-1.72 (-2.44 to -0.98)
Croatia	2134.78（1338.19-3120.64）	113.05（71.48-164.29）	3964.26（2561.92-5738.76）	98.14（63.61-141.95）	-0.46 (-0.53 to -0.39)
Cuba	3362.6（2074.64-5010.29）	184.49（114.8-274.91）	6445.19（4041.31-9483.75）	156.66（98.76-231.06）	-0.54 (-0.67 to -0.41)
Cyprus	2398.05（2000.28-2825.01）	231.98（193.61-272.77）	1739.9（1414.61-2102.2）	113.42（92.5-136.87）	-2.26 (-2.55 to -1.97)
Czechia	32.83（20.27-51.63）	225.91（140.55-352.98）	138.51（84.41-210.7）	197.92（121.72-299.57）	-0.43 (-0.51 to -0.35)
Democratic People's Republic of Korea	21.72（15.92-28.9）	277.65（203.7-369.53）	31.65（21.47-44.81）	289.93（197.24-409.54）	0.14 (0 to 0.27)
Democratic Republic of the Congo	831.76（603.65-1140.37）	192.75（139.71-264.81）	2203.55（1433.63-3251.93）	181.78（118.3-268.21）	-0.15 (-0.56 to 0.26)
Denmark	775.37（643.26-923.36）	127.31（105.54-151.73）	1349.28（981.2-1821.83）	67.05（48.83-90.41）	-2.21 (-3.49 to -0.91)
Djibouti	3905.36（2891.75-5434.75）	130.55（96.14-181.61）	9372.04（6569.07-13060.57）	130.86（92.11-182.06）	0.02

					(-0.29 to 0.33)
Dominica	408.25（321.48-511.02）	115.62（91-144.79）	610.68（450.1-823.73）	79.75（58.76-107.62）	-1.05 (-1.46 to -0.63)
Dominican Republic	51.45（28.99-80.89）	224.91（128.66-351.61）	78.34（43.97-128.55）	146.55（82.98-239.3）	-1.37 (-1.58 to -1.16)
Ecuador	261.57（162.45-385.26）	216.8（136.35-318.07）	498.86（328.71-718.27）	165.8（109.94-238.37）	-0.88 (-0.97 to -0.78)
Egypt	727.38（598.88-867.13）	268.72（221.35-320.73）	509.66（390.99-644.52）	151.47（116.24-191.83）	-1.81 (-2.93 to -0.67)
El Salvador	104.2（60.84-156.11）	315.14（184.89-470.74）	186.65（106.18-280.44）	273.26（156.17-408.81）	-0.44 (-0.65 to -0.23)
Equatorial Guinea	4463.36（2596.9-6575.75）	186.5（109.92-274.02）	5192.94（3595.28-7298.55）	106.58（73.8-149.56）	-1.81 (-1.91 to -1.72)
Eritrea	33.14（22.77-48.04）	89.59（61.16-131.08）	74.98（49.16-110.57）	83.95（54.96-123.93）	-0.2 (-0.61 to 0.21)
Estonia	883.07（730.95-1058.43）	94.32（78.07-113.02）	858.03（687.32-1060.74）	52.42（42.09-64.74）	-1.9 (-2.59 to -1.2)
Eswatini	43531.1（36296.66-52073.48）	414.04（345.24-494.93）	27484.08（21755.09-34033.49）	159.79（126.76-197.82）	-2.95 (-3.49 to -2.41)
Ethiopia	171.34（109.16-255.91）	238.15（151.59-356.5）	242.11（155.17-356.09）	194.47（125.56-284.98）	-0.61 (-0.81 to -0.42)
Fiji	24.04（16.1-34.77）	58.85（39.47-84.96）	61.28（39.94-88.64）	54.88（35.81-79.34）	-0.25 (-0.76 to 0.26)
Finland	3657.33（3251.82-4095.04）	429.03（380.85-480.73）	3061.15（2626.82-3533.39）	379.52（325.63-437.96）	-0.17 (-0.8 to 0.47)
France	25523.53（21381.51-30375.96）	159.3（133.61-189.49）	26505.74（21310.28-32348.53）	111.57（90.13-135.8）	-1.13 (-1.36 to -0.89)
Gabon	705.5（465.39-1048.96）	98.06（64.72-145.74）	2651.85（1592.53-3957.42）	136.58（82.56-203.39）	1.09 (0.95 to 1.24)
Gambia	6832.68（6026.52-7660.02）	339.17（299.02-380.24）	6089.78（5212.59-6980.07）	207.54（178.54-237.7）	-1.57 (-1.85 to -1.29)
Georgia	10.83（7.68-14.82）	284.46（201.79-389.78）	13.46（9.26-19.22）	147.51（101.58-211.86）	-1.94 (-2.29 to -1.6)
Germany	16.9（13.61-20.9）	183.1（147.51-226.43）	19.91（15.47-24.99）	138.02（107.27-173.3）	-0.8 (-2.31 to 0.74)
Ghana	5.69（4.22-7.33）	68.88（50.87-88.88）	7.37（5.67-9.62）	27.33（20.98-35.67）	-2.67 (-3.54 to -1.79)
Greece	771.94（688.47-860.43）	202.64（180.75-225.78）	769.25（625.96-932.05）	58.3（47.52-70.52）	-4.06 (-5.08 to -3.03)
Greenland	430.85（276.71-617.7）	103.52（66.38-148.58）	1046.66（646.8-1600.07）	158.07（98.1-240.81）	1.41 (1.26 to 1.56)
Grenada	84.9（47.67-133.86）	177.13（100.74-277.31）	156.33（90.87-229.5）	190.84（112.51-279.94）	0.25 (0.19 to 0.32)
Guam	65.61（52.1-83.38）	145.19（115.35-184.37）	99.52（69.7-136.25）	122.76（86.25-167.67）	-0.47 (-1.39 to 0.46)
Guatemala	1328.67（791.29-2001.66）	339.85（203.58-511.53）	2265.92（1325.9-3504.33）	278.29（163.45-431.42）	-0.63 (-0.72 to -0.55)
Guinea	335.61（240.57-460.22）	142.66（102.08-195.75）	1372.23（891.63-1955.89）	182.89（118.59-260.64）	0.87 (0.65 to 1.1)
Guinea-Bissau	8338.79（6943.18-10100.32）	416.17（346.37-504.35）	8076.9（6329.98-10054.51）	324.64（253.5-404.53）	-0.77 (-1.4 to -0.13)
Guyana	36.39（30.03-43.99）	98.86（81.61-119.5）	32.9（26.01-40.57）	43.26（34.27-53.31）	-2.52 (-3.06 to -1.97)
Haiti	183609.46（146736.73-224928.42）	334.98（266.25-411.56）	407237.85（346241.38-476331.48）	273.24（232.19-319.64）	-0.65 (-1.12 to -0.17)
Honduras	14476.72（10316.87-18184.9）	129.87（92.27-164.13）	36231.53（24163.69-48149.48）	128.15（85.32-170.42）	-0.04 (-0.11 to 0.02)
Hungary	9904.25（8165.16-11508.09）	319.18（260.03-372.53）	19280.58（16636.27-21984.81）	213.57（183.45-243.69）	-1.29 (-1.41 to -1.17)
Iceland	2656.01（1798.79-3740.04）	291.22（197.46-410.06）	6727.25（4351.06-9672.19）	252.99（164.32-363.65）	-0.41 (-0.65 to -0.17)
India	1170.52（960.73-1393.87）	216.23（177.27-257.66）	821.62（661.57-1013.15）	80.02（64.52-98.66）	-3.08 (-3.67 to -2.48)
Indonesia	893.36（732.6-1078.44）	140.36（114.95-169.43）	1330.92（1056.86-1640.34）	83.39（66.3-102.68）	-1.71 (-2.22 to -1.2)
Iran (Islamic Republic of)	42630.31（39549.8-45692.1）	359.48（333.29-385.48）	26070.82（23154.26-28737.81）	142.99（128.21-157.05）	-2.95 (-3.47 to -2.42)

Iraq	341.27（272.4-420.56）	148.4（118.45-182.81）	627.34（424.89-875.38）	163.5（110.75-228.12）	0.47<br>(0.07 to 0.87)
Ireland	16792.89（15798.91-17674）	77.4（72.63-81.53）	17657.72（15689.55-19076.05）	34.95（31.59-37.57）	-2.49<br>(-2.99 to -2)
Israel	257.41（171.65-367.72）	180.16（120.73-257.51）	653.68（421.92-984）	81.25（52.66-122.01）	-2.48<br>(-2.87 to -2.09)
Italy	5566.01（4979.29-6237.81）	336.97（300.95-378.19）	3355.56（2871.32-3864.64）	139.49（119.33-160.68）	-2.77<br>(-3.36 to -2.17)
Jamaica	1281.16（912.52-1736.94）	133.02（94.82-180.41）	4045.89（2962.26-5180.22）	149.21（109.36-190.79）	0.37<br>(0.23 to 0.51)
Japan	1.07（0.72-1.53）	25.36（16.92-36.38）	2.11（1.33-3.23）	25.62（16.19-39.04）	0.05<br>(0 to 0.09)
Jordan	108.25（86.56-134.24）	180.38（143.98-224）	132.29（97.13-174.36）	49.22（36.13-64.84）	-4.02<br>(-7.34 to -0.58)
Kazakhstan	836.77（658.27-1049.61）	212.61（167.47-266.39）	564.44（414.3-727.22）	92.43（68.08-118.96）	-2.81<br>(-3.9 to -1.71)
Kenya	550.42（352.61-828.66）	217.51（139.68-326.42）	753.35（467.6-1172.16）	143.54（89.52-223.12）	-1.33<br>(-1.4 to -1.26)
Kiribati	1458.53（1215.36-1749.65）	308.4（256.79-370.01）	974.64（760.19-1227.76）	195.59（152.53-246.47）	-1.43<br>(-2.43 to -0.41)
Kuwait	934.34（583.07-1383.29）	350.24（220.75-516.47）	1627.49（1125.68-2305.46）	218.03（150.84-308.92）	-1.45<br>(-1.7 to -1.2)
Kyrgyzstan	251.59（165.79-363.49）	240.17（158.47-346.85）	475.44（299.47-697.99）	339.35（214.32-497.11）	1.19<br>(1.04 to 1.35)
Lao People's Democratic Republic	163.2（102.76-261.34）	114.59（72.15-183.43）	287.27（174.29-453.15）	129.44（78.91-203.58）	0.43<br>(0.14 to 0.72)
Latvia	821.51（541.16-1251.74）	376.95（248.53-573.42）	1966.78（1274.71-2964.87）	354.41（230.53-533.16）	-0.18<br>(-0.48 to 0.14)
Lebanon	1951.62（1611.32-2343.06）	332.15（274.14-399.25）	1868.35（1448.45-2357.47）	263.84（204.4-333）	-0.58<br>(-1.73 to 0.57)
Lesotho	210.77（184.99-240.72）	296.63（260.29-338.57）	156.92（131.86-182.21）	118.86（99.91-138.03）	-3.02<br>(-3.44 to -2.6)
Liberia	1093.34（714.04-1546.12）	179.09（117.41-253.54）	1486.8（970.06-2198.62）	119.77（78.46-176.24）	-1.3<br>(-1.36 to -1.23)
Libya	309.58（211.95-439.81）	66.14（45.32-93.91）	552.19（374.6-829.96）	63.76（43.34-95.43）	-0.09<br>(-0.21 to 0.03)
Lithuania	2142.32（1417.72-3099.38）	202.96（134.01-294.32）	5431.8（3670.98-7658）	154.54（104.47-217.72）	-1<br>(-1.27 to -0.72)
Luxembourg	14.93（9.28-22.35）	157.45（99.3-238.36）	20（13.19-29.07）	58.94（39.02-85.45）	-3.25<br>(-3.51 to -2.99)
Madagascar	601.33（424.97-835.42）	125.56（88.75-174.48）	1177.81（769.78-1758.39）	115.34（75.43-171.8）	-0.24<br>(-0.39 to -0.08)
Malawi	137.9（110.68-167.34）	248.6（199.45-301.74）	115.92（91.41-144.53）	89.19（70.45-111.21）	-3.39<br>(-3.6 to -3.19)
Malaysia	1.81（1.09-2.82）	96.98（58.52-150.56）	3.22（1.9-4.97）	91.01（53.43-141.24）	-0.15<br>(-0.43 to 0.12)
Maldives	133.02（89.23-192.55）	112.82（75.53-163.77）	312.63（190.88-481.71）	122.08（74.72-187.87）	0.28<br>(0.06 to 0.5)
Mali	232.59（201.03-268.34）	252.05（217.83-290.89）	433.33（372.78-497.42）	175.86（151.31-201.95）	-1.28<br>(-3.18 to 0.67)
Malta	12117.45（11598.22-12629.01）	252.51（241.18-263.3）	15041.77（12794.66-17600.51）	98.06（83.5-114.57）	-3.02<br>(-3.39 to -2.65)
Marshall Islands	5.8（3.76-8.88）	97.56（62.96-149.96）	6.78（4.26-10.4）	79.17（50.11-120.94）	-0.68<br>(-0.75 to -0.6)
Mauritania	57.26（37.38-82.72）	633.71（412.63-917.2）	46.44（31.93-66.49）	374.74（257.97-536.71）	-1.7<br>(-1.81 to -1.59)
Mauritius	216.55（150.14-294.91）	171.96（119.14-234.56）	235.74（162.64-336.97）	89.34（61.73-127.6）	-2.19<br>(-2.73 to -1.66)
Mexico	431.24（335.18-550.66）	542.56（421.54-693.21）	730.66（559.48-963.68）	538.35（412.06-709.08）	0.08<br>(-0.2 to 0.35)
Micronesia (Federated States of)	4030.24（2709.9-5731.48）	233.4（156.94-332.36）	9449.03（6005.58-14117.45）	220.87（140.24-329.6）	-0.17<br>(-0.29 to -0.04)
Monaco	1494.51（955.7-2177.57）	212.72（136.7-309.74）	2585.9（1705.53-3729.53）	201.6（133.35-290.12）	-0.15<br>(-0.24 to -0.06)
Mongolia	5681.78（3448.5-8503.28）	201.07（123.4-299.54）	6657.48（4171.73-9846.43）	112.95（71.3-166.86）	-1.85<br>(-1.91 to -1.79)
Montenegro	238.22（167.26-331.35）	285.05（200.16-395.98）	445.51（293.74-644.36）	271.87（180.48-392.46）	-0.14<br>(-0.29 to 0.01)

Morocco	0.6（0.36-0.89）	127.49（77.54-191.16）	0.55（0.33-0.82）	88.08（52.86-133.06）	-1.19<br>(-1.3 to -1.08)
Mozambique	3783.34（2368.82-5718.22）	345.4（216.19-522.5）	7446.49（4796.32-11328.84）	257.22（165.52-391）	-0.93<br>(-1.02 to -0.83)
Myanmar	4299.37（3601.63-5112.37）	166.69（139.73-198.09）	3827.92（3103.41-4638.3）	82.11（66.65-99.42）	-2.37<br>(-2.69 to -2.06)
Namibia	633.49（523.09-760.03）	121.93（100.59-146.41）	542.5（440.98-660.65）	48.32（39.32-58.79）	-2.94<br>(-3.33 to -2.55)
Nauru	261.12（187.31-353.44）	150.79（108.2-204.24）	548.13（379.22-790.18）	95.04（65.67-136.87）	-1.62<br>(-1.84 to -1.4)
Nepal	365.42（227.88-546.85）	111.3（69.91-165.98）	1161.81（678.87-1843.49）	121.92（71.96-192.5）	0.31<br>(0.08 to 0.54)
Netherlands	8715.21（6003.91-11902.76）	162.19（112.67-220.41）	13104.73（9123.84-17947.8）	132.04（92.91-179.66）	-0.67<br>(-0.73 to -0.62)
New Zealand	0.19（0.12-0.28）	66.65（43.08-98.71）	0.17（0.11-0.25）	61.07（39.89-89.88）	-0.29<br>(-0.34 to -0.24)
Nicaragua	1083.52（845.44-1383.89）	455.96（355.15-585.09）	1733.15（1272.6-2337.64）	378.86（279.19-508.88）	-0.63<br>(-0.84 to -0.42)
Niger	1.84（1.18-2.78）	135.76（86.56-204.1）	4.63（3.1-6.61）	81.41（54.2-116.58）	-1.66<br>(-2.14 to -1.18)
Nigeria	812.52（742.68-883.97）	89.86（82.22-97.68）	657（576.87-729.05）	49.7（43.78-55.11）	-1.64<br>(-2.81 to -0.47)
Niue	74.99（47.44-112.9）	101.84（64.66-153.17）	108.44（70.87-160.51）	55.32（35.83-81.92）	-1.92<br>(-2.3 to -1.53)
North Macedonia	40116.59（30279.43-52602.85）	595.18（447.91-781.85）	72599.91（51322.76-99674.88）	529.01（374.24-724.56）	-0.38<br>(-0.47 to -0.29)
Northern Mariana Islands	0.82（0.52-1.22）	70（44.48-103.91）	1.35（0.85-2.11）	52.01（32.54-81.63）	-0.96<br>(-1.15 to -0.78)
Norway	179.31（117.97-264.92）	174.95（115.23-258.02）	315.42（217.22-447.76）	114.08（78.6-161.99）	-1.37<br>(-1.6 to -1.15)
Oman	370.64（321.4-422.1）	209.49（181.48-238.58）	505.77（375.94-635.13）	92.43（68.7-116.09）	-2.65<br>(-3.46 to -1.84)
Pakistan	126.53（74.44-200.13）	67.15（39.8-106.96）	304.43（189.29-486.68）	61.79（37.98-99.14）	-0.24<br>(-0.35 to -0.12)
Palau	408.44（287.62-565.82）	152.86（107.61-211.7）	1398.74（892.67-2090.86）	194.51（124.36-290.55）	0.8<br>(0.33 to 1.28)
Palestine	2017.9（1459.57-2706.83）	146.79（106.16-196.94）	2857.3（1864.98-4198.1）	70.58（46.08-103.7）	-2.24<br>(-3.11 to -1.36)
Panama	3580.62（2938.85-4491.68）	108.86（89.49-136.22）	10293.41（8266.1-12455.09）	104.36（84.11-126.02）	-0.09<br>(-0.18 to 0.01)
Papua New Guinea	24835.85（23614.94-26056.68）	420.7（399.77-441.58）	31454.38（28070.61-34822.12）	320.34（285.89-354.69）	-1<br>(-1.38 to -0.62)
Paraguay	6880.39（5716.69-8335.46）	365.02（303.33-442.1）	5491.49（4352.98-6823.41）	183.31（145.69-227.51）	-2.37<br>(-2.62 to -2.12)
Peru	1283.66（1035.69-1547.77）	274.65（221.62-331.14）	928.25（695.77-1214.02）	100.52（75.39-131.84）	-3.31<br>(-4.75 to -1.86)
Philippines	30.12（19.67-44.91）	371.33（244.58-556.49）	113.12（70.31-177.39）	169.86（106.64-264.54）	-2.49<br>(-3.54 to -1.42)
Poland	9878.21（6521.6-13121.77）	292.1（192.79-387.91）	8751.13（6142.57-11899.15）	71.19（49.95-96.84）	-4.46<br>(-4.68 to -4.23)
Portugal	2089.83（1837.29-2369.7）	350.77（308.2-397.79）	2414.77（2041.87-2837.89）	289.28（244.64-339.83）	-0.57<br>(-2 to 0.89)
Puerto Rico	11326.64（9323.45-13648.15）	297.48（244.57-358.45）	16662.57（13142.39-20782.64）	343.58（270.45-428.91）	0.49<br>(-0.1 to 1.08)
Qatar	81374.2（78182.14-84375.03）	321.59（308.6-333.82）	67004.34（59139.99-74215.42）	197.44（174.4-218.61）	-1.48<br>(-1.89 to -1.07)
Republic of Korea	1039.8（668.28-1541.13）	297.4（191.45-441.09）	1293.24（808.45-2008.56）	170.33（107.22-263.73）	-1.77<br>(-1.83 to -1.7)
Republic of Moldova	9.81（8.38-11.41）	180.89（154.54-210.11）	18.14（14.23-22.86）	198.17（155.97-248.32）	0.29<br>(-0.64 to 1.23)
Romania	32.24（28-36.99）	285.24（247.97-326.66）	72.73（56.27-91.03）	241.54（186.98-301.99）	-0.46<br>(-1.4 to 0.49)
Russian Federation	26.58（22.7-30.73）	282.52（241.36-326.43）	56.67（47.53-67.51）	304.43（255.51-362.22）	0.13<br>(-0.89 to 1.16)
Rwanda	3.6（2.5-5.09）	34.29（23.9-48.67）	5.01（3.43-7.39）	28.85（19.7-42.59）	-0.54<br>(-0.68 to -0.4)
Saint Kitts and Nevis	12.11（8.16-17.03）	264.05（177.76-371.97）	9.27（5.16-14.87）	102.15（56.22-164.77）	-3.36<br>(-3.8 to -2.93)

Saint Lucia	7.53（4.89-11.04）	90.86（58.94-133.11）	13.08（8.57-19.24）	104.17（67.88-153.6）	0.42<br>(0.14 to 0.71)
Saint Vincent and the Grenadines	680.59（444.37-984.99）	108.59（70.95-157.16）	1284.56（874.66-1842.16）	72.39（49.16-103.88）	-1.32<br>(-1.42 to -1.23)
Samoa	489.02（323.2-703.18）	125.2（82.74-179.98）	1330.55（797.59-2013.55）	145.01（87.18-219.57）	0.54<br>(0.15 to 0.92)
San Marino	6859.77（4784.35-9883.06）	447.01（312.11-644.23）	7541.01（5147.36-10463.41）	336.25（229.51-466.45）	-0.89<br>(-1.08 to -0.69)
Sao Tome and Principe	43.86（30.15-60.6）	602.65（414.14-832.87）	64.66（43.96-91.52）	459.31（312.73-649.44）	-0.88<br>(-1.45 to -0.3)
Saudi Arabia	304.01（197.98-450.95）	122.2（79.48-181.38）	585.72（373.05-904.45）	135.97（86.81-209.2）	0.35<br>(0.28 to 0.43)
Senegal	556.02（454.92-672.45）	214.02（174.96-258.97）	626.71（494.48-780.76）	57.56（45.36-71.75）	-4.13<br>(-5.08 to -3.18)
Serbia	3041.49（2171.36-4089.7）	376.58（268.92-506.64）	3078.25（2115.43-4382.17）	237.24（163.03-337.55）	-1.51<br>(-1.56 to -1.45)
Seychelles	885.47（733.87-1054.3）	277.48（229.77-330.63）	870.32（691.37-1082.95）	154.2（122.78-191.64）	-2.11<br>(-3.11 to -1.1)
Sierra Leone	14.42（8.32-22.6）	100.29（59.57-156.52）	28.4（17.69-43.15）	80.54（50.25-122.36）	-0.72<br>(-0.87 to -0.57)
Singapore	573.37（338.01-891.96）	238.39（142.14-369.6）	1287.18（782.41-1953.04）	181.73（111.48-274.55）	-0.86<br>(-0.95 to -0.77)
Slovakia	4548.3（3517.35-6593.51）	182.59（141.39-264.09）	10041.64（8738.28-11447.37）	172.66（150.02-196.9）	-0.23<br>(-0.62 to 0.16)
Slovenia	753.99（458.95-1155.13）	233.65（142.35-358.12）	741.16（438.54-1160.93）	178.9（106.37-279.24）	-0.84<br>(-0.98 to -0.7)
Solomon Islands	33252.62（27354.54-39852.9）	453.92（373.46-543.93）	23195.4（18041.95-28950.19）	197.66（154.02-246.17）	-2.72<br>(-3.24 to -2.19)
Somalia	1327.96（936.63-1858.64）	107.85（75.89-151.54）	4024.08（2241.59-6410.96）	111.87（62.84-177.78）	0.09<br>(-0.45 to 0.63)
South Africa	3053.4（1752.76-5210.97）	277.59（158.57-478.41）	4554.39（2751.91-7060.13）	216.68（131.1-335.62）	-0.8<br>(-0.84 to -0.75)
South Sudan	32.15（23.44-43.29）	106.38（77.68-142.95）	72.54（45.3-108.16）	91.77（57.38-136.95）	-0.39<br>(-1.13 to 0.35)
Spain	1281.97（1081.61-1505.93）	64.29（54.32-75.43）	1067.2（850.41-1308.57）	37.7（30.07-46.22）	-1.6<br>(-2.95 to -0.23)
Sri Lanka	2166.06（1796.17-2590.29）	167.06（138.75-199.63）	1566.98（1238.35-1938.51）	69.06（54.8-85.34）	-2.96<br>(-3.46 to -2.45)
Sudan	1015.92（730.26-1424.15）	171.18（122.97-240.54）	2079.78（1436.31-2903.31）	135.67（94.53-188.02）	-0.82<br>(-1.19 to -0.45)
Suriname	2990.73（2486.68-3573.54）	144.81（120.22-173.36）	3923.33（3097.99-4917.81）	69.18（54.66-86.69）	-2.26<br>(-2.73 to -1.79)
Sweden	582.43（427.75-767.38）	173（127.49-227.93）	684.94（456.36-962.69）	100.1（65.37-141.22）	-1.75<br>(-2.12 to -1.38)
Switzerland	9466.01（6794.63-13142.41）	237.03（169.71-328.7）	19371.14（12771.09-28775.96）	136.81（90.3-203.18）	-1.76<br>(-2.05 to -1.46)
Syrian Arab Republic	34.91（21.79-53.65）	127.95（80.89-195.26）	124.13（78.35-187.48）	114.17（72.08-172.78）	-0.36<br>(-0.47 to -0.24)
Taiwan (Province of China)	177.38（115.97-263.39）	127.17（83.25-188.57）	669.04（397.89-1004.48）	153.54（92.38-230.06）	0.61<br>(0.32 to 0.91)
Tajikistan	0.15（0.09-0.24）	79.89（49.03-125.71）	0.11（0.07-0.17）	55.3（34.83-86.16）	-1.2<br>(-1.26 to -1.14)
Thailand	4.54（2.88-7.15）	70.58（44.55-111.08）	5.96（3.67-9.2）	62.07（38.27-95.89）	-0.4<br>(-0.72 to -0.08)
Timor-Leste	183（159.92-210.06）	174.36（152.46-199.99）	350.48（257.51-454.81）	134.16（98.76-173.92）	-0.93<br>(-1.07 to -0.8)
Togo	2007.5（1325.9-2920.1）	326.6（216.02-474.24）	4164.1（2514.3-6488.82）	245.45（148.6-381.23）	-0.93<br>(-1.11 to -0.75)
Tokelau	17725.64（11499.74-27125.7）	433.79（282.42-661.16）	26358.79（17663.69-37813.47）	223.09（149.59-319.72）	-2.16<br>(-2.57 to -1.74)
Tonga	650.36（574.95-724.9）	272.18（239.62-304.3）	582.89（449.64-755.23）	116.54（90.14-150.74）	-2.67<br>(-3.18 to -2.14)
Trinidad and Tobago	0.71（0.48-1.03）	83.7（55.85-120.95）	0.88（0.58-1.29）	68.34（45.05-100.13）	-0.66<br>(-0.7 to -0.61)
Tunisia	1963.92（1317.93-2763.61）	256.46（172.5-360.34）	3234.76（2068.01-4897.78）	197.72（127.15-297.22）	-0.86<br>(-1.02 to -0.69)
Turkey	30611.84（25149.43-36663.17）	302.67（248.53-362.68）	20130.92（12985.52-29611.71）	187.93（121.34-276.3）	-1.52<br>(-2.07 to -0.98)

Turkmenistan	77.22（43.96-123.45）	218.81（126.67-347.23）	343.98（225.65-501.46）	136.26（91.04-195.61）	-1.65 (-2.99 to -0.29)
Tuvalu	18148.87（17436.64-18817.18）	152.32（146.38-157.91）	15581.22（14496.59-16488.86）	93.16（87.08-98.44）	-1.62 (-2.04 to -1.2)
Uganda	2858.73（1851.12-4203.19）	214.79（139.63-315.45）	4112.4（2573.33-6631.28）	141.94（89.24-227.13）	-1.34 (-1.43 to -1.25)
Ukraine	20.12（13.93-28.93）	172.07（163.68-179.09）	29.7（18.68-44.36）	104.79（97.64-110.82）	-1.84 (-2.31 to -1.36)
United Arab Emirates	71697.41（68146.89-74641.29）	200.69（138.97-288.4）	82267.44（76582.04-87039.51）	117.18（73.6-175.31）	-1.66 (-1.83 to -1.49)
United Kingdom	2984.56（2431.32-3634.73）	575.4（468.73-700.8）	2153.99（1728.23-2651.53）	311.65（250.32-383.45）	-2.01 (-2.15 to -1.87)
United Republic of Tanzania	3221.65（2668.66-3821.05）	226.73（188.13-268.3）	2446.72（1868.7-3140.26）	77.28（59.17-98.71）	-3.51 (-4.23 to -2.79)
United States of America	4.89（2.95-7.61）	73.07（43.94-113.8）	10.84（6.86-16.52）	58.23（36.69-88.97）	-0.72 (-0.86 to -0.59)
United States Virgin Islands	3755.1（3329.8-4186.35）	335.07（296.53-373.77）	9261.19（6809.4-12372.6）	242.7（178.89-323.45）	-1.16 (-1.48 to -0.83)
Uruguay	7499.34（5043.05-10911.01）	147.75（99.66-214.78）	19471.09（12321.93-29131.98）	157.71（100.79-234.62）	0.22 (0.09 to 0.35)
Uzbekistan	1790.45（1065.12-2851.85）	307.91（182.42-490.82）	4439.23（2555.96-6832.01）	282.73（162.81-436.42）	-0.25 (-0.37 to -0.14)
Vanuatu	797.56（526.7-1148.09）	246.6（162.94-355.42）	1700.13（840.33-3774.33）	224.42（112.87-488.55）	-0.29 (-0.48 to -0.09)
Venezuela (Bolivarian Republic of)	1219.51（827.29-1719.08）	244.72（166.44-344.32）	1907.77（1307.97-2708.57）	232.24（159.59-328.93）	-0.11 (-0.34 to 0.12)
Viet Nam	214695.24（179808.97-254988.32）	295.88（247.28-351.69）	429895.63（379221.77-494084.68）	245.19（216.08-281.95）	-0.59 (-0.9 to -0.27)
Yemen	275857.16（247845.2-300780.59）	224.91（201.85-245.11）	536503.97（478646.19-602011.58）	160.4（142.92-179.92）	-1.08 (-1.2 to -0.96)
Zambia					
Zimbabwe	3543.93（2893.5-4333.66）	150.2（122.47-183.8）	5691.06（4216.3-7464.68）	79.32（58.8-104.01）	-2.03 (-2.58 to -1.48)

Abbreviations: SDI: Sociodemographic Index; ASDR: age-standardised disability-adjusted life years rate; AAPC=average annual percentage change; CI = confidence interval; UI = uncertainty interval.

Table S4. Changes in incident number according to population-level determinants and causes from 1990 to 2021.

Location	Overall difference [a]	Change due to population-level determinants			Percent change of Aging	Percent change of Population	Percent change of Epidemiological change	Overall percent change
		Aging [b]	Population [c]	Epidemiological change [d]				
Global	59453.5	31.52	83136.68	-23714.71	0.04	111.83	-31.9	79.97
High SDI	9647.96	-303.86	19607.28	-9655.47	-1.19	76.91	-37.87	37.84
High-middle SDI	12135.27	-168.17	20359.84	-8056.4	-0.75	90.89	-35.96	54.17
Low SDI	2887.52	9.3	3501.44	-623.22	0.3	113.78	-20.25	93.83
Low-middle SDI	12259.64	63.32	13513.47	-1317.16	0.66	140.61	-13.7	127.56
Middle SDI	22453.74	347.21	23506.54	-1400.01	2.54	172.17	-10.25	164.46

a.Change in incident number between year 2021 and 1990;

b.Change in incident number due to change in the age structure;

c.Change in incident number due to change in population number;

d.Change in incident number due to epidemiologic changes. Epidemiologic changes refer to the incident number change when age structure and population hold constant.

Abberrations: SDI = Socio-demographic index.

Table S5. Changes in death number according to population-level determinants and causes from 1990 to 2021.

Location	Overall difference [a]	Change due to population-level determinants			Percent change of Aging	Percent change of Population	Percent change of Epidemiological change	Overall percent change
		Aging [b]	Population [c]	Epidemiological change [d]				
Global	28713.21	1160.41	56578.58	-29025.78	2.14	104.43	-53.57	53
High SDI	613.42	276.06	8578.14	-8240.78	2.23	69.23	-66.51	4.95
High-middle SDI	2536.67	334.01	13621.01	-11418.35	1.99	81.01	-67.91	15.09
Low SDI	2579.03	42.23	3402.73	-865.93	1.38	111.15	-28.28	84.24
Low-middle SDI	10149.53	206.42	12679.42	-2736.31	2.19	134.75	-29.08	107.87
Middle SDI	12803.84	735.88	18967.93	-6899.97	5.92	152.72	-55.55	103.09

a.Change in death number between year 2021 and 1990;

b.Change in death number due to change in the age structure;

c.Change in death number due to change in population number;

d.Change in death number due to epidemiologic changes. Epidemiologic changes refer to the death number change when age structure and population hold constant.

Abberrations: SDI = Socio-demographic index.

Table S6. Changes in DALYs number according to population-level determinants and causes from 1990 to 2021.

Location	Overall difference [a]	Change due to population-level determinants			Percent change of Aging	Percent change of Population	Percent change of Epidemiological change	Overall percent change
		Aging [b]	Population [c]	Epidemiological change [d]				
Global	559325.68	-19829.92	1245992.93	-666837.33	-1.63	102.32	-54.76	45.93
High SDI	-2801.46	-4842.83	182892.73	-180851.36	-1.79	67.66	-66.91	-1.04
High-middle SDI	30924.53	-8669.01	303474.12	-263880.58	-2.26	78.99	-68.69	8.05
Low SDI	54762.5	-768.55	77366.23	-21835.18	-1.09	109.22	-30.83	77.31
Low-middle SDI	215200.39	-2489.19	284300.24	-66610.65	-1.16	132.42	-31.03	100.24
Middle SDI	260646.82	-1776.96	412891.7	-150467.93	-0.64	149.68	-54.55	94.49

a.Change in DALYs number between year 2021 and 1990;

b.Change in DALYs number due to change in the age structure;

c.Change in DALYs number due to change in population number;

d.Change in DALYs number due to epidemiologic changes. Epidemiologic changes refer to the DALYs number change when age structure and population hold constant.

Abberrations: SDI = Socio-demographic index.
